# Phylogenetic, clinical, pathological and epidemiological characterization of feline coronavirus infections in cats, in Istanbul

**DOI:** 10.3389/fvets.2025.1645884

**Published:** 2025-10-10

**Authors:** Gulay Yuzbasioglu Ozturk, Abdullah Kayar, Ozge Erdogan Bamac, Hasan Emre Tali, Ismail Egemen Ozkan, Sajid Umar, Ozge Aydin, Utku Yusuf Cizmecigil, Onur Iskefli, Alper Bayrakal, Fikriye Suzer Turuncoglu, Bilge Kaan Tekelioglu, Eduardo Berriatua, Chris Helps, Aydin Gurel, Nuri Turan, Juergen A. Richt, Huseyin Yilmaz, Aysun Yilmaz

**Affiliations:** ^1^Department of Pathology, Veterinary Faculty, Istanbul University-Cerrahpasa, Istanbul, Türkiye; ^2^Department of Internal Medicine, Veterinary Faculty, Istanbul University-Cerrahpasa, Istanbul, Türkiye; ^3^Department of Virology, Veterinary Faculty, Istanbul University-Cerrahpasa, Istanbul, Türkiye; ^4^Global Health Research Center (GHRC), Duke Kunshan University, Wuzhong, China; ^5^Division of Natural & Applied Sciences (DNAS), Duke Kunshan University, Wuzhong, China; ^6^Pets-In Veterinary Clinic, Istanbul, Türkiye; ^7^Department of Virology, Veterinary Faculty, Cukurova University, Adana, Türkiye; ^8^Departamento de Sanidad Animal, Facultad de Veterinaria, Regional Campus of International Excellence "Campus Mare Nostrum", Universidad de Murcia, Murcia, Spain; ^9^Langford Veterinary Services, University of Bristol, Bristol, United Kingdom; ^10^Department of Diagnostic Medicine/Pathobiology, College of Veterinary Medicine, Kansas State University, Manhattan, KS, United States

**Keywords:** feline coronavirus, cats, Türkiye, serology, haematology, PCR, phylogenetic, histopathology

## Abstract

**Introduction:**

Feline coronavirus (FCoV) is a widespread viral infection affecting domestic and wild cats globally, with higher prevalence in young cats and multi-cat environments.

**Methods:**

In this study, a total of 208 clinical samples (blood, fecal, ascitic fluid, pleural fluid, tissue) were collected between January 2018 and January 2020 from diseased cats. Clinical and demographic data were recorded, and hematobiochemical and molecular detection analyses were performed.

**Results:**

A total of 189 blood samples (90.9%) were found seropositive for FCoV, while 79 fecal samples (38%) were found positive for FCoV RNA by real-time RT-qPCR. No significant association was found between FCoV-RNA positivity and age or gender, while a significant association was found with crossbreed cats (*p <* 0.05). Notable clinical signs included weight loss (47%), dullness (44%), respiratory distress (16%), vomiting (13%), ascites (13%), epileptic fits (13%), diarrhea (6%), and fever (5%). Fever, depression, diarrhea, and ascites were significantly more common in PCR-positive cats than in PCR-negative cats (*p <* 0.05). The relationship between FCoV-RNA positivity and hematobiochemical indices was variable. Elevated monocyte and neutrophil levels were observed in 51 and 29% of cases, respectively. Additionally, elevated ALT activity and bilirubinemia were detected in 19 and 28% of cats, respectively. Partial S gene nucleotide analysis showed a deletion of multiple nucleotides in all sequences obtained in the present study. Interestingly, these deletions were absent in all reference strains belonging to FCoV type 2. Among 68 FCoV strains, 42 formed a separate cluster with the reference strain (AY307020) during phylogenetic analysis. This cluster was further divided into several small sub-clusters. Several unique recombinant events and recombination signals were observed among partial S1 gene sequences. Notable histopathological findings included fibrinous serositis and pyogranulomatous inflammation in vital organs.

**Discussion:**

This study provides comprehensive information on FCoV infections among cats in Turkey. The findings could significantly contribute to understanding the hematobiochemical manifestations, epidemiology, and risk factors associated with FCoV, ultimately aiding in the development of better prevention and treatment strategies. A continuous molecular surveillance program is required to understand the evolution and emergence of virulent strains of FCoV to design new antiviral therapies and vaccines.

## Introduction

Feline coronavirus (FCoV) infects both domesticated and wild cats (African lion, mountain lion, leopard, cheetah, jaguar, lynx, serval, caracal, European wildcat, sand cat and Pallas’s cat) worldwide (1). FCoV exists in two distinct forms: feline enteric coronavirus (FECV), which replicates in the intestinal tract, and feline infectious peritonitis virus (FIPV), the pathogenic variant responsible for feline infectious peritonitis (FIP) (2, 3). Cats that develop FIP experience severe systemic illness with high mortality, resulting in economic burdens from hospitalization and treatment, as well as profound emotional distress for their owners.

FCoV is an enveloped, positive-sense, single-stranded RNA virus with a non-segmented genome and helical symmetry. It belongs to the order *Nidovirales*, family *Coronaviridae*, subfamily *Coronavirinae*, and genus *Alphacoronavirus* (4, 5). The genome is about 29 Kb in size and contains 11 open reading frames (ORFs). These encode 4 major structural proteins: nucleocapsid (N), membrane (M), envelope (E) and spike (S), (6) and 7 nonstructural proteins, which includes five accessory proteins (3a, 3b, 3c, 7a and 7b) and two replicase proteins (1a and 1b) (2, 7). FCoVs are further divided into two serotypes (I and II) based on antigenicity. Serotype I virus is of feline origin and serotype II virus appears to have arisen from the recombination of FCoV serotype I with canine coronavirus (1, 8–10). The two pathotypes (FECV and FIPV), exist in both serotypes I and II (3, 11). Serotype I FCoV does not grow well in cell culture and grows only in macrophage derived cell lines (12). Antisera to canine coronavirus (CCV) has a weak response to FCoV I. In contrast, serotype II FCoV grows well in cell culture and reacts with antisera to CCV (13).

FCoV is primarily shed in the feces of healthy carrier cats and transmitted through the fecal-oral route. Transmission occurs most efficiently in multi-cat environments, where the infection rate is significantly higher compared to single-cat households. FCoV demonstrates notable environmental stability, remaining infectious on fomites for 3–7 weeks, making contaminated objects potential transmission vehicles (1, 9, 14, 15). Additionally, persistently infected asymptomatic carriers play a crucial epidemiological role, as most shed virus either continuously or intermittently for months to years (15–18).

FIP as a disease in cats was first reported in 1966 (19). After this report, FIPV and FECV were suggested as aetiological agents in FIP between 1978 and 1981 (12, 20). FECV is similar to FIPV in terms of antigenicity, but different in pathogenicity with FIPV being more virulent and may cause death within a few weeks after infection (2, 19, 21). In addition to differences in pathogenicity *in vivo*, the two biotypes have different cellular tropisms (2). In general, during natural infections, FECV has tropism for mucosal epithelial cells or mesenteric lymph nodes (2, 3, 12), whereas FIPV infects mainly macrophage cell lines as well as lymphocytes, plasma cells and neurocytes (2, 3, 22, 23). FECV has been shown to be present systemically in monocytes of healthy cats (15, 24, 25).

The prevalence of FCoV infections may be up to 90% in multi-cat environments and 10–60% in house-hold cats worldwide (26–31). Primary FCoV infection occurs in enterocytes and passes to blood by monocyte-associated viremia (21, 32, 33). While approximately 20% of FECV-infected cats experience viral mutations, only 12%–13% of these cases progress to FIP. This progression depends on key host and viral factors, including viral virulence and the nature of the host’s immune response particularly strong, rapid cellular immune reactions that may contribute to immunopathological effects (29). The development of FIP correlates strongly with several risk factors including stress, concurrent infections, immunity, high population density. The disease shows particular predilection for young cats aged 3–16 months, when maternal antibody protection wanes and the juvenile immune system remains vulnerable (9, 16, 25, 28, 29). At present, two clinical forms of FIP are well documented: a ‘wet’ or effusive form (polyserositis and vasculitis) and a ‘dry’ or non-effusive form (pyogranulomatous lesions in organs) (21, 32). Infiltration of ascites from tissues into the pleural cavity, peritoneal cavity and pericardial cavity is the most prominent manifestation of ‘wet’ FIP, while lethargy, anorexia, weight loss and fever refractory to antibiotics are common and non-specific signs of FIP (29, 32, 34, 35). Non-effusive FIP is characterized by granuloma formation involving the central nervous system, eyes and abdominal organs (especially kidneys, liver, mesenteric lymph nodes and intestinal wall), and does not produce body cavity effusion (21, 34).

While most FCoV-infected cats remain asymptomatic, clinical cases typically present with gastrointestinal manifestations, most commonly vomiting and diarrhea. The clinical presentation of FIP varies significantly between its effusive (‘wet’) and non-effusive (‘dry’) forms. However, both forms typically share several hallmark clinical features including anorexia, lethargy, dehydration, icterus, and neurological manifestations such as ataxia, paresis, and hyperesthesia (21, 36). Neurological signs are more frequent in non-effusive FIP (3). In addition, fever, weight loss, diarrhoea, ocular lesions and kidney disorders are also prominent in cats with non-effusive FIP. In effusive FIP, abdominal distention, ascites, pleural and pericardial effusion, dyspnea are the main clinical signs of disease (35–38).

Currently, no commercially available vaccine exists for FIP. Vaccine development has been hindered by significant genetic variability among circulating FCoV strains (3). Furthermore, effective treatment of FIP remains challenging due to the complex pathogenesis of the disease. A 3C-like protease inhibitor (GC376) was used in the experimental treatment of FIP cats in 2016. This was subsequently developed to treat naturally occurring FIP, but recurrences of disease were observed (39). A nucleoside analog (GS-441524) was found to be a safe and effective treatment of FIP, and interfered with virus replication (21, 40, 41). The protease inhibitors have been used in clinical treatment recently, but their usage is limited because of the high price, long treatment period and relatively low cure rate (3, 21).

In the absence of effective vaccines or reliable treatments for FIP, establishing accurate diagnostic protocols for FCoV infection becomes crucial for case management and disease control. However, FIP diagnosis remains challenging, requiring comprehensive evaluation of multiple parameters including clinical presentation, hematological and biochemical abnormalities, serological testing, virological analysis, and histopathological examination. FIP remains a frequent diagnostic challenge in feline medicine, with misdiagnosis rates exceeding 40% in first-opinion practice (3, 29, 42–46). This is important since some veterinarians start corticosteroids after diagnosis (35). This might increase the manifestation of latent herpesvirus and toxoplasma if present in cats with FIP (3). Therefore, the aforementioned diagnostic parameters need to be collected and evaluated together (3, 9, 45, 47, 48). For virological analyses, virus isolation and molecular tests like RT-PCR and real time RT-PCR are used and are very helpful, particularly when using abdominal or pleural fluid or tissue biopsy or aspirates (1, 45, 49). Results from RT-PCR tests should be evaluated together with clinical findings and postmortem samples (1, 29, 40, 44, 45, 49). Also there are rapid tests and ELISAs for antigen detection in faeces to monitor cats carrying FCoV as well as to check antibody response. However, serological tests have low specificity and sensitivity and may fail to detect recent infections and cross-reactions occur between FIPV and low pathogenic FECV strains (9, 45). While hematological and biochemical alterations in FIP lack pathognomonic specificity, a constellation of the findings (e.g., CBC abnormalities, Serum protein changes, Acute phase reactants, liver function tests, kidney function tests, Hyperbilirubinemia) should raise clinical suspicion. These parameters exhibit 78% combined diagnostic accuracy when ≥4 abnormalities are present, though none are definitive alone (3, 29, 36, 47). Histopathological analysis is considered the gold standard for diagnosing FIP. Currently, definitive confirmation of FIP can only be achieved through immunohistochemistry to detect FCoV antigen in biopsy samples, affected tissues, and macrophages present in effusion fluids (3).

Currently, there is limited data on the epidemiology, clinicopathological features, and molecular characteristics of FCoVs in Türkiye. This study aimed to investigate FCoV seroprevalence, perform molecular and phylogenetic analyses, and evaluate associations with signalment (including habitat), clinical and biochemical parameters, and histopathological findings in cats from Istanbul, Türkiye. Additionally, we sought to generate novel data on circulating FCoV strains in Istanbul, which could serve as a reference for future prevention and control strategies against FCoV infections.

## Materials and methods

### Study population, clinical examination and sampling

This study was conducted on clinically ill cats suspected of FCoV infection based on serological analyses. The animals were referred to the Department of Internal Medicine at Veterinary Faculty of Istanbul University-Cerrahpasa between January 2018 and January 2020. Data on each cat’s gender, breed, age, and origin (household, pet shop, or stray) were recorded. The study population consisted of 208 cats aged 2 months to 15 years, with a nearly equal gender distribution (103 females, 105 males). The cohort included 46 purebred and 162 mixed-breed cats. Based on origin, 165 were household cats, 37 were strays, and 13 were obtained from pet shops, while the origin of 5 cats remained undocumented.

All cats were clinically examined for the presence of fever, behavioral changes like depression and clinical signs related to organ systems, mainly respiratory (wheezing, dyspnea and abnormal lung sounds), gastrointestinal (mouth lesions, anorexia, vomitus, diarrhoea, weight loss, abdominal distension and/or ascites), circulatory (lymphoadenopathy, anaemia, icterus), urinary, ocular lesions (conjuctivitis, keratic precipitates, uveitis, hyphema, iridocyclitis, chorioretinitis) and central nervous system (epileptic seizures, ataxia) were recorded. Cats were enrolled as suspected FIP/FCoV cases if they demonstrated at least two of the following clinical signs: (1) systemic signs including prolonged fever (>48 h unresponsive to antibiotics), >10% body weight loss, or persistent lethargy (>7 days); (2) effusive disease manifestations (ascites, pleural effusion, or pericardial effusion); or (3) non-effusive disease features such as ocular signs (uveitis, retinal vasculitis), neurological abnormalities (ataxia, seizures), or palpable abdominal masses (mesenteric lymphadenopathy). Cases were further supported by characteristic laboratory abnormalities including A: G ratio <0.6, hyperglobulinemia (>5 g/dL), or lymphopenia (<1.5 × 10^3^/μL). Cats were excluded from the study if they met any of the following conditions: (1) had alternative definitive diagnoses including positive FeLV/FIV status or confirmed cases of toxoplasmosis, bacterial peritonitis, or neoplasia (diagnosed via cytology or histopathology); (2) received prior FIP-specific treatment with antiviral or immunosuppressants (e.g., corticosteroids) within 14 days preceding enrollment; or (3) presented with an incomplete diagnostic workup, specifically the absence of paired effusion/serum samples for effusive cases or missing baseline hematology and biochemistry profiles.

During the study, faecal swabs from all cats and abdominal (ascites) and/or pleural effusions from cats having “Wet” FIP were taken. Blood samples were also taken with and without EDTA from the cephalic vein for haematological and biochemical analyses and to detect antibodies to FCoV in all cats and feline immunodeficiency virus (FIV) and feline leukemia virus (FeLV) antigen in 36 cats as described below.

In addition to the study cohort, postmortem examinations and histopathological analyses were conducted on 43 cats that died with a presumptive diagnosis of FIP. These cases were referred to the Department of Pathology from veterinary clinics throughout Istanbul, Türkiye. The inclusion of this additional cohort was designed to provide comprehensive pathological and molecular characterization of circulating FCoV strains in Istanbul. Complete tissue sampling was performed on all 43 cases, including brain, heart, lung, liver, kidneys, spleen, intestine, and mesenteric lymph nodes. When present, abdominal (ascitic) and/or pleural effusions were also collected and submitted to the Department of Virology for molecular analysis.

#### Serum sample analysis for FCoV, FIV and FeLV detection

Sera from the 208 cohort cats were analysed for the presence of antibodies to FCoV by using rapid Immunochromatographic assay to detect antibodies against FCoV (Bionote, Hwaseong, South Korea). The Antigen Rapid FCoV Ab Test Kit is a chromatographic immunoassay for the qualitative detection of FCoV antibodies in feline whole blood, serum, or plasma. In addition, 36 cats for FIV antibodies and FeLV antigen (IDEXX, Snap FIV/FeLV Combo Plus test kit) as described by the manufacturer.

### Haematological and biochemical analyses

Blood samples from cats were analyzed for a complete blood haemogram–histogram (15 parameters); specifically, total white blood cell count (WBC), red blood cell counts (RBC), haemoglobin, haematocrit, mean corpuscular volume (MCV), mean corpuscular haemoglobin concentration (MCHC), red cell distribution width (RDW), reticulocyte, lymphocyte, neutrophil, monocyte, eosinophil, basophil, platelet and mean platelet volume (MPV) as described by the manufacturer (IDEXX Procyte Dx Reagent Kit).

Samples from cats were also analyzed for comprehensive blood biochemistry (14 parameters); alanine aminotransferase (ALT), alkaline phosphatase (ALP), total bilirubin, total protein, albumin (alb), globulin (glob), albumin/globulin ratio, blood urea nitrogen (BUN), creatinine, glucose, phosphorus (P), calcium (Ca), gama-glutamyl transferase (GGT) and cholesterol were measured using commercial kits (IDEXX Catalyst Chem 15 and IDEXX Catalyst Chem 17).

### Postmortem examination and histopathology

Systematic necropsies were performed on 43 deceased cats submitted to the Department of Pathology. Gross lesions consistent with FIP including body cavity effusions, serosal thickening, nodular lesions, and mesenteric lymphadenomegaly were thoroughly assessed. Tissue samples from the brain, heart, lungs, liver, kidneys, spleen, intestines, and mesenteric lymph nodes were fixed in 10% buffered formalin for 24 h, paraffin-embedded, sectioned at 3–4 μm, and stained with hematoxylin and eosin (H&E). Additionally, liver, brain, and intestinal samples, along with pleural and/or abdominal effusions (when present), were stored at −80 °C for subsequent FCoV detection via real-time RT-PCR.

Histopathological evaluations were conducted by two board-certified veterinary pathologists. FIP-associated lesions such as fibrinous serositis, granulomatous inflammation, and vasculitis/perivasculitis in the kidneys, liver, lungs, brain, and intestines—were documented and correlated with real-time RT-PCR results, gross pathology, and clinical history.

### Virological analyses

#### Extraction of RNA, cDNA synthesis and real time RT-PCR

For the extraction of RNA, about 25 mg of tissue was mixed with glass beads and homogenised (Bullet Blender, Next Advance). Total RNA was then extracted from the homogenised tissues, ascites and pleural effusions and faecal swabs (incubated in lysis buffer 15 min) by using a commercial RNA extraction kit (Cat No:12183018A, Invitrogen) as described by the manufacturer. The concentration of RNA was measured using a NanoDrop spectrophotometer (NanoDrop 1000c, Thermo Scientific, Waltham, USA).

In order to generate complementary DNA (cDNA), the total RNA was reverse transcribed by using a commercial cDNA synthesis kit (High Capacity cDNA reverse transcription kit, Cat. No: 4368814, Applied Biosystems™) as described by the manufacturer.

For FCoV detection via real-time RT-qPCR, previously published primers and TaqMan probes (Table 1), targeting the membrane-nucleocapsid gene junction of FCoV, were used (17). Real time RT-qPCR was first optimised by using different amounts of primers, template DNA and reagents. An optimised real time RT-qPCRs consisted of 12.5 μL Maxima Probe/ROX qPCR Master Mix (Thermo Scientific 2X Master Mix, Cat. No: K0231), 1 μL forward primer (10 μM), 1 μL reverse primer (10 μM), 1 μL of TaqMan probe (10 μM), 0.5 μL MgCl_2_ (50 mM), 2 μL cDNA and 7 μL nuclease free water. All amplifications were performed in a real time PCR machine (Thermo Fisher Scientific, Applied Biosystems, StepOnePlus, USA). Cycling conditions were 95 °C for 10 min followed by 45 cycles of 95 °C for 10 s, 56 °C for 15 s and 72 °C for 15 s. For all reactions, positive and negative controls were included. Nuclease-free water was used as the negative control in place of template. Positive controls consisted of cDNA from samples previously confirmed as FCoV-positive through sequence analysis at the Department of Virology, Veterinary Faculty of Istanbul University-Cerrahpasa.

**Table 1:** Primers and probe used in this study for FCoV detection.

PCR	Primer and probe	Target gene	Size	Position	Reference
RT-PCR	Primer F: CTTGGTGCACGTCTTGAATC	Spike gene	642 bp	23106–23125	This study
	Primer R: ACTCAACGC TTCACCCTG			23730 23747	
Real time RT-PCR	Primer F: AGCAACTACTGCCACRGGAT	Memhrane-nucleocapsid gene	171 bp	26655–26674	Dye et al. (17)
	Primer R: GGAAGGTTCATCTCCCCAGT			26807–26826	
	Taqman Probe: AATGGCCACACAGGGACAACGC			26781–26802	

#### Sequencing and phylogenetic analysis

Samples positive for FCoV by real time RT-qPCR were subjected to further RT-PCR to allow DNA sequencing analysis. A pair of primers designed in this study were used to amplify a 642 bp region of the S gene of FCoV (Table 1). The RT-PCR was first optimized by using different amounts of template and primers. An optimized 25 μL PCR consisted of 12.5 μL Maxima Probe/ROX qPCR Master Mix (Thermo Scientific 2X Master Mix, Cat. No: K0231, Lithuania), 1 μL (10 μM) of each primer, 0.5 μL MgCl_2_ (25 mM) (Thermo Scientific, Cat. No: R0971, Lithuania), 2 μL of cDNA and 8 μL nuclease free water. Nuclease-free water was used as a negative control in place of template. Positive controls were obtained from samples previously submitted to the Department of Virology, Veterinary Faculty of Istanbul, and confirmed to be FCoV positive by RT-qPCR and sequencing. All amplifications were done in a qPCR instrument (Q-Tower, Analytik Jena, Germany). Cycling conditions were 95 °C for 10 min followed by 40 cycles of 94 °C for 1 min, 55 °C for 1 min, 72 °C for 1 min and finally 72 °C for 10 min. Amplification products were separated in 1.5% agarose gels containing 0.25 μg/mL nucleic acid staining solution (INTRON Biotechnology, RedSafe, Cat No: 21141) by electrophoresis. Amplified PCR products were sent to a commercial company for Sanger sequencing (MedSantek, Istanbul, Türkiye).

For phylogenetic analysis, nucleotide sequences of the partial S gene PCR products were manually edited in Chromas Pro and aligned using the MAFFT version 7 (online version) (50). Data available in the National Centre for Biotechnology Information (NCBI) were used to compare the genotypic relationship between FCoV strains from this study and other FCoV strains submitted from other countries. The maximum Likelihood: RAxML method was used to construct the phylogenetic tree with 1,000 Bootstrap replicates using MegAlign Pro Software (DNASTAR). Circle format of the phylogenetic tree was reconstructed by FigTree (Version 1.4.5 pre). MegAlign Pro Software (DNASTAR) was used to determine the percentage identity between the FCoV strains. Partial S gene sequences of 68 field strains of FCoV detected in this study were submitted to GenBank. Reference strains for FCoV type 1 (JN183882.1, FJ938060.1, MH817484.1, LC742526.1, PP908788.1, KU215428.1, DQ848678.1) and FCoV type 2 (NC_002306.3, PP526173.1, KC461237.1, OQ311323.1, JQ408981) were used for comparative analysis. In addition, canine and porcine coronavirus reference strains were also included for analysis (GQ477367.1, DQ811787.1).

#### Recombination analysis

Gene recombination analysis was performed using RDP5 (v.5.45) in the aligned genome sequences. We used seven methods in RDP5 including RDP, GENECONV, 3Seq, Chimera, Bootscan, SiScan and MaxChi (51).

### Statistical analyses

Data were analyzed using GraphPad-Prism (Version 10.2.3). Fisher’s exact test (52) was used to compare the proportion of FCoV seropositive cats and RT-qPCR positive cats according to cat demographic and habitat variables and the proportion of clinical signs in FCoV seropositive/PCR positive and seronegative/PCR negative cats. In our primary analysis, we reported unadjusted *p*-values to maintain full transparency of all statistical results in context of effect sizes(e.g., odds ratios with 95% confidence intervals), and biological plausibility.

Biochemical and haematological results were categorized as being within (normal), above (high) or below (low) reference values (53). The independent relationship between FCoV PCR results status, and a cat’s demographic and habitat variables was further investigated using logistic regression analysis (54). FCoV was the binary outcome variable (seropositive or seronegative and PCR-positive and PCR-negative). The explanatory variables included those associated at *p* < 0.05 with FCoV serological and PCR results status in the univariable analysis; age, examination year and the variable reflecting habitat, outdoor access and contact with other cats. All variables were modeled as categorical, with the exception of age. Age was categorized into five levels ranging from 2 months to 15 years old. Model parameters were estimated using the maximum likelihood estimation method and significance was taken for alpha less than 5% for a double sided test.

## Results

### Prevalence of FCoV

FCoV RNA was detected in faecal swabs by real time RT-qPCR in 79 of 208 cats analyzed, with an estimated prevalence of 38% (31.4–44.6%, 95% confidence interval). The results included 44/101 cats (44%) analyzed in 2018 and 35/107 (33%) in 2019 (*p* > 0.05) (Table 2). Seroprevalence of FCoV was 90.9% (189/208, 87.0–94.8, 95% confidence interval) (Table 3). Seroprevalence was similar in 2018 and 2019, and was not associated with cat demographic and habitat variables (*p* > 0.05).

**Table 2:** The estimated FCoV PCR prevalence according to year and cat demographic variables.

Variables	Parameters	*N*	Negative	Positive	% Positive	95%CI		*p* value	Statistically significant (*p* < 0.05)
						Lower	Upper		
Study year	2018	101	57	44	44	34	53	0.1176	No
	2019	107	72	35	33	24	42		
Gender	Female	103	64	39	38	28	47	>0.9999	No
	Male	105	65	40	38	29	47		
Breed	Cross breed	162	107	55	34	27	41	0.038	Yes
	Pure breed	46	22	24	52	38	67		
Age (years)	1 = 2 to <6 month	39	20	19	49	33	64	0.4714	No
	2 = 6 month to <1 age	64	39	25	39	27	51		
	3 = 1 to <2 age	48	31	17	35	22	49		
	4 = 2 to <3 age	20	13	7	35	14	56		
	5 = 3 to <4 age	10	8	2	20	−5	45		
	6 = 4 to <6 age	17	13	4	24	3	44		
	7 = 6 to <11 age	9	4	5	56	23	88		
	8 = >11 age	1	1	0	0	0	0		

**Table 3:** The estimated FCoV seroprevalence according to year and cat demographic variables.

Variables	Parameters	*N*	Negative	Positive	% Positive	95%CI		*p* value	Statistically significant (*p* < 0.05)
						Lower	Upper		
Study year	2018	101	8	93	92	87	97	0.634	No
	2019	107	11	96	90	84	95		
Gender	Female	103	9	94	91	86	97	>0.9999	No
	Male	105	10	95	90	85	96		
Breed	Cross breed	162	17	145	90	85	94	0.2572	No
	Pure breed	46	2	44	96	90	102		
Age (years)	1 = 2 to <6 month	39	7	32	82	70	94	0.090	No
	2 = 6 month to <1 age	64	4	60	94	88	100		
	3 = 1 to <2 age	48	1	47	98	94	102		
	4 = 2 to <3 age	20	3	17	85	69	101		
	5 = 3 to <4 age	10	1	9	90	71	109		
	6 = 4 to <6 age	17	1	16	94	83	105		
	7 = 6 to <11 age	9	2	7	78	51	105		
	8 = >11 age	1	0	1	100	100	100		

Amongst 36 cats tested for FIV and FeLV, 12 cats were found to be positive for FCoV RNA. In 12 FCoV PCR positive cats, only 1 cat for FIV and 1 cat for FeLV were positive.

The relationship between FCoV-RNA positivity (infection), signalment, and habitat variables for the cats analyzed in this study are presented in Tables 2, 4–6. No significant association was found in FCoV RNA positivity with age and gender while significant association was found in FCoV-RNA positivity with crossbreed cats (*p* < 0.05) (Table 2). When the habitat of the cats considered, the percentage of FCoV-RNA positive cats was 38% (63/166) in household and 32% (12/37) in street cats (Table 4). The FCoV-RNA positivity in the category of origins was as follows; 42.2% (19/45) in home raised cats, 61.5% (8/13) in petshop cats and 66.9% (97/145) in street cats. Amongst these cats, 30.7% (35/114) of the cats cohabitating with other cats and 23% (14/61) had access outdoors and were cohabitating with other cats. When the presence of FCoV-RNA is considered, the origin, access to outdoor and cohabitation with other cats were statistically significant (*p* < 0.05) (Table 4).

**Table 4:** The estimated FCoV PCR prevalence according to cat’s FCoV antibody status origin and habitat variables.

Variables	Parameters	*N*	Negative	Positive	% Positive	95%CI		*p* value	Statistically significant (*p* < 0.05)
						Lower	Upper		
FCoV serodiagnosis	FCoV antibody positive	189	116	73	39	32	46	0.6267	No
	FCoV antibody negative	19	13	6	31.6	11	52		
Home/	Household	166	103	63	38.0	31	45	0.5768	No
	Street	37	25	12	32.4	17	48		
Origin	Home raised	45	26	19	42.2	28	57	0.0127	Yes
	Pet shop	13	5	8	61.5	35	88		
	Street	145	48	97	66.9	59	75		
Outdoor access	No	142	81	61	43.0	35	51	0.0071	Yes
	Yes	61	47	14	23.0	12	34		
Cohabitating with other cats	No	89	49	40	44.9	35	55	0.0412	Yes
	Yes	114	79	35	30.7	22	39		

**Table 5:** The estimated FCoV PCR prevalence according to cat habitat variables.

Variables	Level	Origin			*N*	Negative	Positive	% Positive	95%CI		*p* value	Statistically significant (*p* < 0.05)
		Home raised	Pet shop	Street					Lower	Upper		
Home; outdoor access; cohabitating with other cats	Street; yes; yes	–	–	37	37	25	12	32	17	48	0.0058	Yes
	House; no; no	23	7	59	89	49	40	45	35	55		
	House; no; yes	18	4	31	53	32	21	40	26	53		
	House; yes; yes	4	2	18	24	22	2	8	−3	19		

**Table 6:** The estimated risk of FCoV PCR-positivity according to year and cats age and habitat variables.

Variables	Level	Odds ratio	95%CI		*p* value	Statistically significant (*p* < 0.05)
			Lower	Upper		
Study year	2018	1	–	–	–	
	2019	0.7436	0.4015	1.371	0.3432	No
Age (years)	1 = 1 to <6 month	1	–	–	–	
	2 = 6 to <11 month	0.6322	0.2661	1.487	0.2942	No
	3 = 11 to <23 month	0.6217	0.2473	1.541	0.3065	No
	4 = 23 to <60 month	0.3668	0.141	0.921	0.0352	Yes
	5 = 60 to <180 month	1.389	0.3098	6.502	0.6662	No
Home; outdoor access; cohabitating with other cats	House; no; no	1	–	–	–	
	House; no; yes	0.8218	0.4009	1.668	0.5882	No
	House; yes; yes	0.1086	0.0165	0.411	0.0045	Yes
	Street; yes; yes	0.5943	0.251	1.353	0.223	No

Data about the serological status, signalment and habitat variables of the cats analysed in this study is given in Table 7. No significant association was found in FCoV seroprevalence with breed, age and gender. When the habitat of the cats was considered, the percentage of FCoV positive cats was 91% (41/45) in home raised, 92.1% (105/114) in cats cohabitating with other cats and 93.4% (57/61) in cats that had access to outdoors. These findings were not statistically significant.

**Table 7:** The estimated FCoV seroprevalence according to cat origin and habitat variables.

Variables	Level	*N*	Negative	Positive	% Positive	95%CI		*p* value	Statistically significant (*p* < 0.05)
						Lower	Upper		
Home	Household	166	15	151	91.0	87	95	0.7559	No
	Street	37.0	4.0	33.0	89.2	79	99		
Origin	Home raised	45	4	41	91.1	83	99	>0.9999	No
	Pet shop	13.0	1.0	12.0	92.3	78	107		
	Street	145	14	131	90.3	86	95		
Outdoor access	No	142.0	15.0	127.0	89.4	84	94	0.4413	No
	Yes	61	4	57	93.4	87	100		
Cohabitating with other cats	No	89.0	10.0	79.0	88.8	82	95	0.4714	No
	Yes	114	9	105	92.1	87	97		

Logistic regression analysis confirmed the independent relationship of FCoV-RNA status of household cats (habitat), access to outdoor and having contact with other cats (Table 5). In addition, the presence of FCoV RNA in cats between the age of 23 months to 60 months was statistically significant (*p* < 0.05) (Table 6).

### Clinical-signs

Table PCR-PCR3 presents the percentage of 79 FCoV-PCR positive cats exhibiting specific clinical signs. Clinical signs and associated percentages in these cats were weight loss (47%), depression/dullness (44%), respiratory distress (16%), vomiting (13%), ascites (13%), abdominal distention with no ascites (11%), epileptic fits (13%), ocular lesions (11%), urinary signs (9%), stomatitis (8%), diarrhoea (6%) and fever (5%). Fever, depression, diarrhoea, and Ascites were significantly more common in PCR-positive than in PCR-negative cats (*p* < 0.05) (Table 8).

**Table 8:** The estimated prevalence of clinical signs according to cat’s FCoV RT-qPCR status.

Clinical findings	FCoV RT-PCR positive					FCoV RT-PCR negative					*p* value	Statistically significant (*p* < 0.05)
	*N*	No. affected	% Positive	95%CI		N	No. affected	% Negative	95%CI			
				Lower	Upper				Lower	Upper		
Fever	79	5	6	6	7	129	22	17	16	18	0.0325	Yes
Depression or dullness	79	35	44	42	47	129	82	64	62	66	0.0093	Yes
Diarrhea	79	5	6	6	7	129	23	18	17	19	0.0209	Yes
Stomatitis	79	6	8	7	8	129	12	9	9	10	0.8018	No
Ocular signs	79	9	11	10	13	129	16	12	11	13	>0.9999	No
Weight loss	79	37	47	44	50	129	73	57	54	59	0.1985	No
Vomiting	79	10	13	11	14	129	28	22	20	23	0.1385	No
Abdominal distention	79	9	11	10	13	129	25	19	18	21	0.1760	No
Ascites	79	10	13	11	14	129	32	25	23	26	0.0493	Yes
Pleural effusion	79	3	4	3	4	129	17	13	12	14	0.0291	Yes
Respiratory sign	79	13	16	15	18	129	32	25	23	26	0.1694	No
Dysuria	79	7	9	8	10	129	8	6	6	7	0.5824	No
Epilepsy	79	10	13	11	14	129	9	7	6	8	0.2154	No

Amongst the 189 FCoV seropositive cats, 57% were depressed, 52% had weight loss, 21% showed respiratory distress, 19% had ascites and 15% abdominal distension, vomiting and diarrhea were present in 17 and 12%, respectively, 12% had ocular signs and 7%–8% had stomatitis, urinary signs, pleural effusion and epileptic fits (Table 9). The percentage of cats with pleural effusion was significantly lower in seropositive compared to seronegative animals *p* < 0.05 (Table 9).

**Table 9:** The estimated prevalence of clinical signs according to cat FCoV antibody status.

Clinical findings	FCoV seropositive					FCoV seronegative					*p* value	Statistically significant (*p* < 0.05)
	*N*	No. affected	% Positive	95%CI		*N*	No. affected	% Negative	95%CI			
				Lower	Upper				Lower	Upper		
Fever	189	25	13	12	14	19	2	11	8	13	>0.9999	No
Depression or dullness	189	107	57	55	58	19	10	53	47	58	0.8105	No
Diarrhea	189	23	12	11	13	19	5	26	22	31	0.1475	No
Stomatitis	189	16	8	8	9	19	2	11	8	13	0.6723	No
Ocular signs	189	23	12	11	13	19	2	11	8	13	>0.9999	No
Weight loss	189	99	52	51	54	19	11	58	52	63	0.8103	No
Vomiting	189	33	17	16	19	19	5	26	22	31	0.3529	No
Abdominal distention	189	28	15	14	16	19	5	26	22	31	0.1939	No
Ascites	189	35	19	17	20	19	6	32	27	37	0.2219	No
Pleural effusion	189	13	7	6	7	19	7	37	32	42	0.0006	Yes
Respiratory signs	189	39	21	19	22	19	6	32	27	37	0.2564	No
Dysuria	189	15	8	7	8	19	0	0	0	0	0.3703	No
Epilepsy	189	16	8	8	9	19	3	16	13	19	0.3919	No

### Hematological and biochemical findings

The clinical hematology and biochemistry results for FCoV-RNA positive cats (*n* = 72–73) are summarized in Table 10. Notably, 24% of cats exhibited high WBC counts, with elevated monocyte and neutrophil levels in 51 and 29% of cases, respectively, while 40% had low eosinophil counts. Additionally, 25% of cats had low RBC counts, 47% had reduced hemoglobin levels, and 52% had low hematocrit. Thrombocyte counts were abnormally low in 27% of cats. Furthermore, 97% of 37 cats tested had a decreased albumin/globulin ratio, while low BUN and creatinine levels were observed in 41 and 24% of cases, respectively (Table 10). Elevated ALT activity was detected in 19% of cats, and bilirubinemia was present in 28%. There were no significant differences between FCoV-RNA positive and negative cats in the proportion of abnormally high or low values of haematological and biochemical parameters analyzed except in the percentage of cats with low albumin/globulin ratio with was significantly higher among FCoV-RNA positive cats (Table 10).

**Table 10:** The estimated prevalence of abnormal levels of haematological and biochemical clinicopathological parameters according to cat’s FCoV RT-qPCR status.

Variables	FCoV RT-PCR positive					FCoV RT-PCR negative					*p* value	Statistically significant (*p* < 0.05)
	*N*	No. affected	%95CI	95%CI		*N*	No. affected	%95CI	95%CI			
				Lower	Upper				Lower	Upper		
Hematology												
High WBC	72	17	24	21	26	119	44	37	35	39	0.0572	No
Low WBC	72	3	4	4	5	119	8	7	6	7	0.5395	No
High lymphocytic count	72	6	8	7	9	118	14	12	11	13	0.6268	No
Low lymphocytic count	72	3	4	4	5	118	5	4	4	5	>0.9999	No
High red blood cell count	73	1	1	1	2	120	1	1	1	1	>0.9999	No
Low red blood cell count	72	18	25	23	27	120	35	29	27	31	0.6178	No
High hemoglobin	73	1	1	1	2	120	0	0	0	0	0.3782	No
Low hemoglobin	73	34	47	44	49	120	52	43	41	46	0.7653	No
High hematocrit	73	2	3	2	3	120	1	1	1	1	0.5581	No
Low hematocrit	73	38	52	49	55	120	57	48	45	50	0.556	No
High mean corpuscular volume (MCV)	73	0	0	0	0	120	4	3	3	4	0.2992	No
Low mean corpuscular volume (MCV)	73	17	23	21	25	120	16	13	12	14	0.0801	No
High mean corpuscular hemoglobin volume (MCHC)	73	9	12	11	14	120	10	8	8	9	0.4559	No
Low mean corpuscular hemoglobin volume (MCHC)	73	0	0	0	0	120	2	2	2	2	0.5272	No
High red cell distribution width (HRDW)	72	23	32	29	35	120	35	29	27	31	0.7461	No
Low red cell distribution width (LRDW)	72	0	0	0	0	120	0	0	0	0	>0.9999	No
High reticulocyte number	73	3	4	4	5	117	6	5	5	6	>0.9999	No
Low reticulocyte number	73	1	1	1	2	117	6	5	5	6	0.2531	No
High monocytes	72	37	51	48	54	118	69	58	56	61	0.3686	No
Low monocytes	72	0	0	0	0	118	0	0	0	0	>0.9999	No
High neutrophil granulocytes	72	21	29	27	32	117	49	42	40	44	0.0893	No
Low neutrophil granulocytes	72	2	3	2	3	117	6	5	5	6	0.7124	No
High eosinophil granulocytes	72	2	3	2	3	116	4	3	3	4	>0.9999	No
Low eosinophil granulocytes	72	29	40	37	43	116	58	50	48	52	0.2294	No
High basophil granulocytes	70	3	4	4	5	115	2	2	2	2	0.3683	No
Low basophil granulocytes	70	3	4	4	5	115	5	4	4	5	>0.9999	No
High platelet count	73	3	4	4	5	120	2	2	2	2	0.3684	No
Low platelet count	73	20	27	25	30	120	24	20	19	21	0.2886	No
High mean platelet volume (MPV)	73	6	8	7	9	120	9	8	7	8	>0.9999	No
Low mean platelet volume (MPV)	73	0	0	0	0	120	2	2	2	2	0.5272	No
Proteins												
High albumin	37	0	0	0	0	73	0	0	0	0	>0.9999	No
Low albumin	37	4	11	9	12	73	10	14	12	15	0.7697	No
High globulin	37	14	38	34	42	73	40	55	52	58	0.109	No
Low globulin	37	0	0	0	0	73	0	0	0	0	>0.9999	No
High alb./glob. Ratio	37	1	3	2	3	73	5	7	6	8	0.6616	No
Low alb./glob. Ratio	37	36	97	97	98	73	8	11	10	12	<0.0001	Yes
High total protein	36	12	33	30	37	73	23	32	29	34	>0.9999	No
Low total protein	36	1	3	2	3	73	4	5	5	6	>0.9999	No
Kidney												
High BUN	37	3	8	7	9	74	17	23	21	25	0.0682	No
Low BUN	37	15	41	37	45	74	23	31	29	34	0.3969	No
High creatinine	37	1	3	2	3	74	7	9	8	10	0.265	No
Low creatinine	37	9	24	21	27	74	21	28	26	31	0.8211	No
Liver												
High ALT	37	7	19	16	21	74	11	15	13	16	0.594	No
Low ALT	37	1	3	2	3	74	0	0	0	0	0.3333	No
High ALP	37	2	5	5	6	73	5	7	6	8	>0.9999	No
Low ALP	37	12	32	29	36	73	21	29	26	31	0.826	No
High bilirubin	18	5	28	23	33	34	14	41	37	45	0.3819	No
Low bilirubin	18	0	0	0	0	34	0	0	0	0	>0.9999	No

Overall, the hematological and serum biochemical profiles of FCoV-seropositive cats closely resembled those of the FCoV-RNA positive cats described above. Specifically, the percentages of seropositive cats with elevated white blood cells, monocyte, and neutrophil counts were 31, 56, and 37%, respectively, while 28, 48, and 44% had decreased RBC, hematocrit, and hemoglobin levels, respectively. Additionally, 23% exhibited thrombocytopenia. Notable biochemical abnormalities among seropositive cats included a low albumin/globulin ratio (40%), elevated bilirubin levels (35%), increased ALT and ALP activity (16 and 7%, respectively), as well as low and high BUN levels (35 and 17%) and low and high creatinine levels (28 and 8%) (Table 11). In comparison, significantly fewer FCoV-seronegative cats exhibited low albumin/globulin ratios and low BUN levels, while a higher proportion had elevated WBC counts (with none showing low WBC) (*p* < 0.05) (Table 11). Results of clinical hematology and biochemistry among seropositive cats are shown in Table 11. FCoV seroprevalence was significantly associated with high reticulocyte counts and high platelet counts (*p* < 0.05).

**Table 11:** The estimated prevalence of abnormal levels of haematological and biochemical clinicopathological parameters according to cat’s FCoV antibody status.

Variables	FCoV seropositive					FCoV seronegative					*p* value	Statistically significant (*p* < 0.05)
	*N*	No. affected	%95CI	95%CI		*N*	No. affected	%95CI	95%CI			
				Lower	Upper				Lower	Upper		
Hematology												
High WBC	178	56	31	30	33	13	6	46	39	53	0.3575	No
Low WBC	178	11	6	6	7	13	0	0	0	0	>0.9999	No
High lymphocytic count	177	18	10	9	11	13	2	15	12	19	0.6316	No
Low lymphocytic count	177	7	4	4	4	13	1	8	6	10	0.439	No
High red blood cell count	180	2	1	1	1	13	0	0	0	0	>0.9999	No
Low red blood cell count	180	51	28	27	30	13	2	15	12	19	0.5205	No
High hemoglobin	180	1	1	1	1	13	0	0	0	0	0.1395	No
Low hemoglobin	180	79	44	42	46	13	7	54	47	61	0.5689	No
High hematocrit	180	3	2	2	2	13	0	0	0	0	>0.9999	No
Low hematocrit	180	86	48	46	50	13	9	69	63	75	0.1594	No
High mean corpuscular volume (MCV)	180	4	2	2	2	13	0	0	0	0	>0.9999	No
Low mean corpuscular volume (MCV)	180	30	17	16	18	13	3	23	18	28	0.469	No
High mean corpuscular hemoglobin volume (MCHC)	180	16	9	8	9	13	3	23	18	28	0.1226	No
Low mean corpuscular hemoglobin volume (MCHC)	180	2	1	1	1	13	0	0	0	0	>0.9999	No
High red cell distribution width (HRDW)	179	52	29	28	31	13	6	46	39	53	0.2175	No
Low red cell distribution width (LRDW)	179	0	0	0	0	13	0	0	0	0	>0.9999	No
High reticulocyte number	177	6	3	3	4	13	3	23	18	28	0.0167	Yes
Low reticulocyte number	177	7	4	4	4	13	0	0	0	0	>0.9999	No
High monocytes	177	100	56	55	58	13	6	46	39	53	0.567	Yes
Low monocytes	177	0	0	0	0	13	0	0	0	0	>0.9999	No
High neutrophil granulocytes	176	65	37	35	39	13	6	46	39	53	0.5595	No
Low neutrophil granulocytes	176	8	5	4	5	13	0	0	0	0	>0.9999	No
High eosinophil granulocytes	175	6	3	3	4	13	0	0	0	0	>0.9999	No
Low eosinophil granulocytes	175	79	45	43	47	13	8	62	55	68	0.2673	No
High basophil granulocytes	172	5	3	3	3	13	0	0	0	0	>0.9999	No
Low basophil granulocytes	172	8	5	4	5	13	0	0	0	0	>0.9999	No
High platelet count	180	3	2	2	2	13	2	15	12	19	0.0374	Yes
Low platelet count	180	42	23	22	25	13	2	15	12	19	0.736	No
High mean platelet volume (MPV)	180	15	8	8	9	13	0	0	0	0	0.604	No
Low mean platelet volume (MPV)	180	2	1	1	1	13	0	0	0	0	>0.9999	No
Proteins												
High albumin	103	0	0	0	0	7	0	0	0	0	>0.9999	No
Low albumin	103	13	13	12	14	7	1	14	10	19	>0.9999	No
High globulin	103	52	50	48	53	7	2	29	21	36	0.4382	No
Low globulin	103	0	0	0	0	7	0	0	0	0	>0.9999	No
High alb./glob. Ratio	103	6	6	5	6	7	0	0	0	0	>0.9999	No
Low alb./glob. Ratio	103	41	40	37	42	7	3	43	34	52	>0.9999	No
High total protein	102	33	32	30	35	7	2	29	21	36	>0.9999	No
Low total protein	102	4	4	4	4	7	1	14	10	19	0.2871	No
Kidney												
High BUN	104	18	17	16	19	7	2	29	21	36	0.6075	No
Low BUN	104	36	35	32	37	7	2	29	21	36	>0.9999	No
High creatinine	104	8	8	7	8	7	0	0	0	0	>0.9999	No
Low creatinine	104	29	28	26	30	7	1	14	10	19	0.6718	No
Liver												
High ALT	104	17	16	15	18	7	1	14	10	19	>0.9999	No
Low ALT	104	0	0	0	0	7	1	14	10	19	0.0631	No
High ALP	103	7	7	6	7	7	0	0	0	0	>0.9999	No
Low ALP	103	30	29	27	31	7	3	43	34	52	0.4263	No
High bilirubin	51	18	35	32	38	1	1	100	100	100	0.3654	No
Low bilirubin	51	0	0	0	0	1	0	0	0	0	>0.9999	No

### Necropsy findings

**Figure 1:**
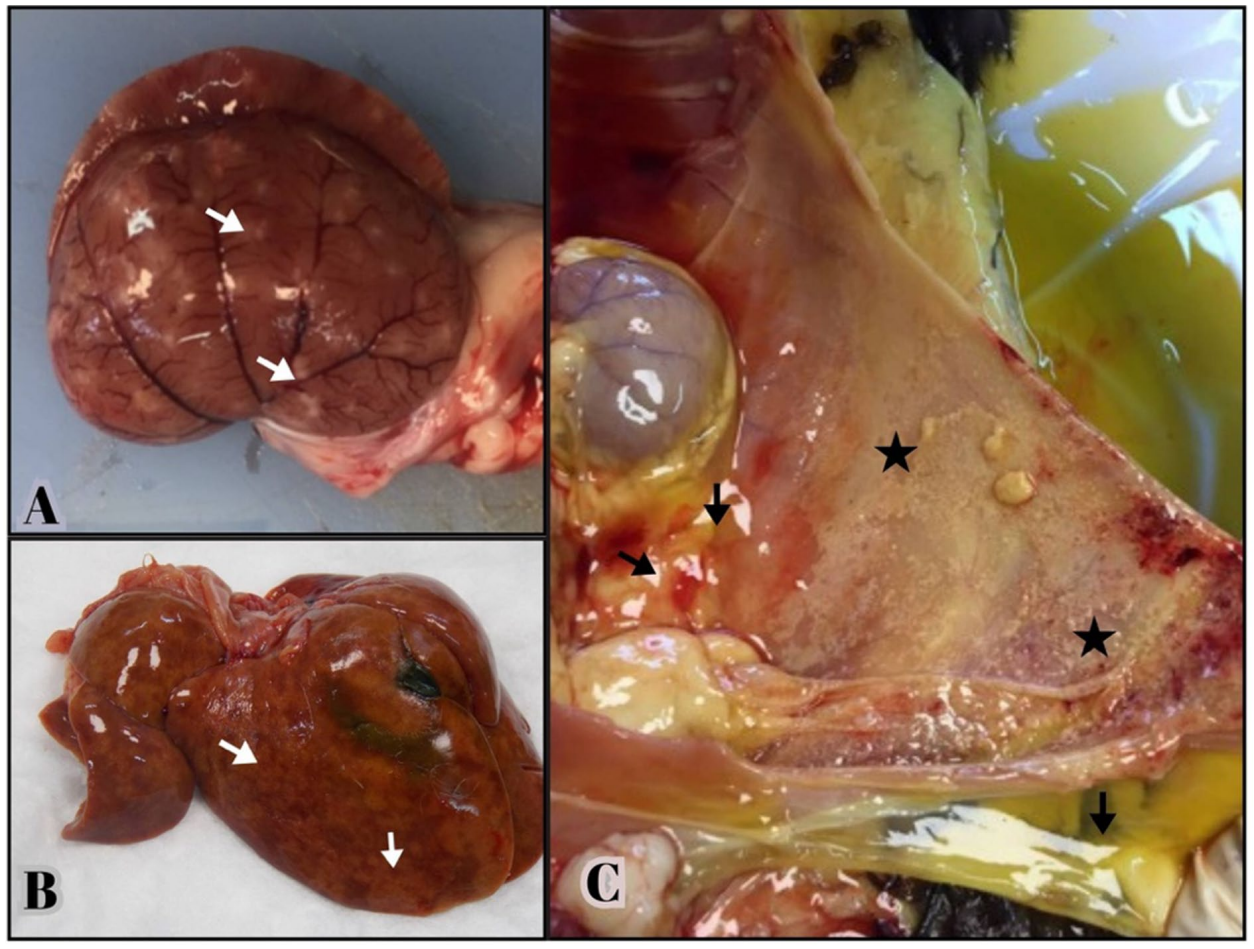
Gross lesions from necropsied cats positive for FCoV RNA by qRT-PCR and histologic lesions consistent with FIP. **(A,B)** Multifocal granulomas (arrows) are evident on the capsular surface of the kidney, extending into the renal parenchyma and on the surface of the liver. **(C)** Yellowish, viscous fluid is visible in the abdominal cavity (arrows). The serosal surface of the abdominal wall consists of small yellow—tan nodules and fibrin deposition consistent with pyogranulomatous inflammation (stars).

Necropsy revealed fibrinous, yellowish effusion in the body cavities of 19 out of the 43 deceased cats (44.1%). Abdominal effusion was present in 16 cats (37.2%), while thoracic effusion was observed in 9 cats (20.9%), with 6 cats (13.9%) exhibiting both. Abdominal and thoracic effusions (13.9%). Fibrin deposition was particularly prominent in the liver and peritoneum of 16 cats, often accompanied by severe adhesions between abdominal organs. Multifocal granulomatous lesions were detected in 17 cats, primarily the liver and kidneys, followed by the lungs (37.2%) (Figures 1A,B).

### Histopathologic findings

Lesions characteristic of FIP, including fibrinous serositis, granulomatous/pyogranulomatous inflammation and vasculitis/ perivasculitis in various organs such as the liver, brain, intestines, lungs, kidneys, heart, spleen and pancreas were evaluated in histopathologic examination of all 43 cats (Supplementary Figure 1). Fibrinous serositis was most commonly observed in the liver (*n* = 13), lungs (*n* = 11) and intestines (*n* = 5) (Supplementary Figures 1A,B). Granulomatous/ pyogranulomatous inflammation was most prominent in the liver (*n* = 16), followed by the kidneys (*n* = 6) and intestines (*n* = 6) (Supplementary Figure 1C). Vasculitis/ perivasculitis was identified most frequently in the liver (*n* = 21), in the lungs (*n* = 9), brain (*n* = 6) and kidneys (*n* = 4) (Supplementary Figure 1D). Other consistent brain lesions included cerebral meningitis (*n* = 13) and lymphoplasmacytic meningoencephalitis (*n* = 2). Based on histopathologic findings, the liver appeared to be the most affected organ. Other significant microscopic findings included moderate to severe lymphoid depletion in the spleen, lymphoplasmacytic nephritis, interstitial pneumonia, and pancreatitis. Correlation of histopathological findings and PCR results has been shown in Supplementary Table 1.

### Phylogenetic analysis

**Figure 2:**
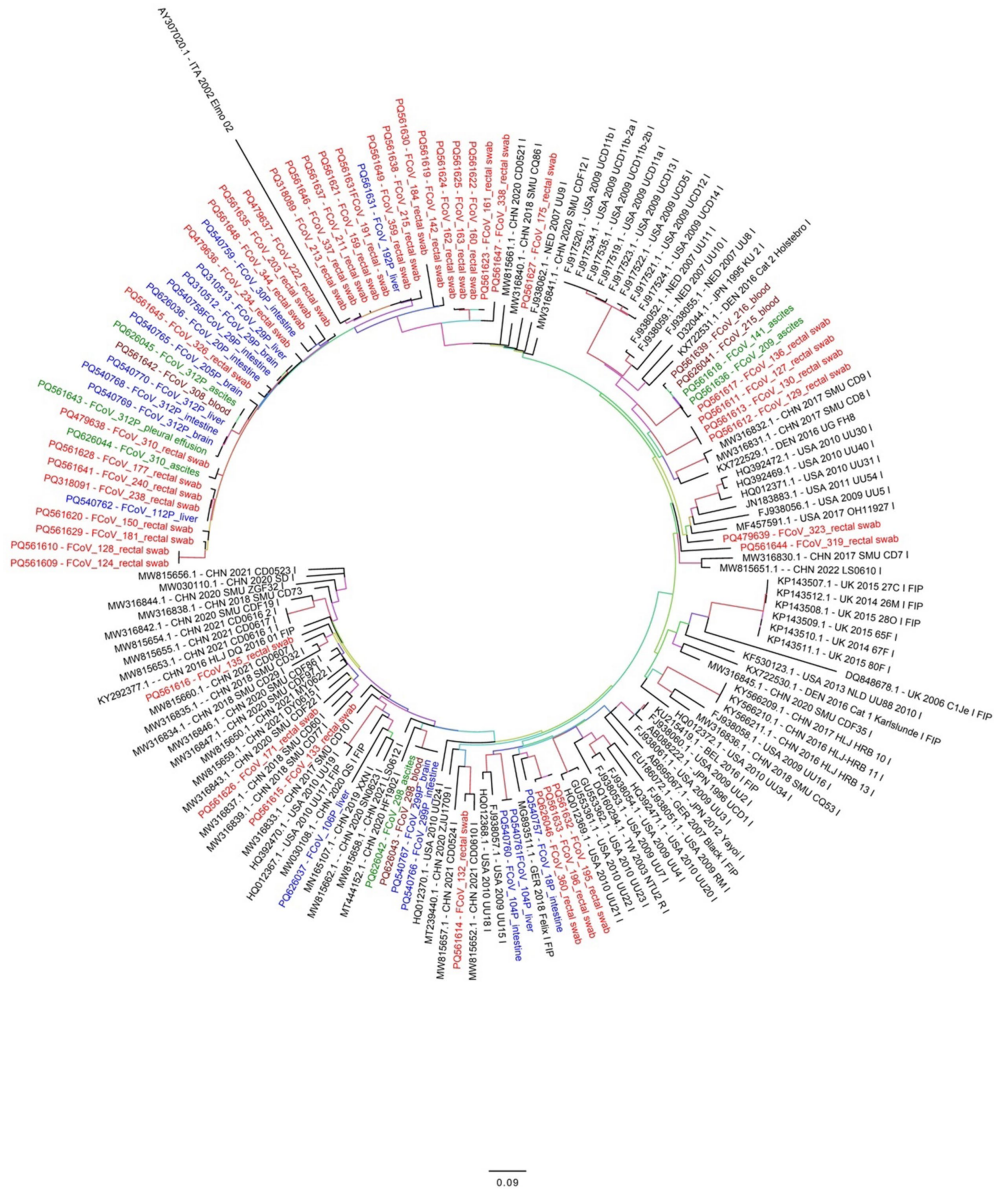
Phylogenetic analysis based on the FCoV S gene nucleotide sequences. The obtained 68 FCoV strains in this study are marked with different colors (red or blue or green). Red colored names indicate FCoV detected from cat rectal swabs while blue colored names indicate FCoV detected from brain, liver or intestine of infected cats in this study. Similarly, green colored names indicates FCoV detected from ascites fluid or effusions and dark brown color indicate FCoV detected from blood samples of infected cats. The phylogenetic tree was inferred using the maximum likelihood (ML) method and each ML tree was tested 1,000 times with a Bootstrap test to estimate the branch support.

From the 79 samples found to be positive by real time RT-qPCR, the expected 642 bp partial S1 gene amplicon was seen after conventional PCR and gel electrophoresis and successfully sequenced from 68 samples. Phylogenetic analysis revealed that all 68 FCoV strains belonged to type I FCoV (FCoV-I). A phylogenetic tree, based on the partial S1 gene sequence was generated (Figure 2). Comparative analysis showed a nucleotide identity of 80%–98% among all partial S gene sequences in this study (Supplementary Table 2).

For evolutionary analysis, a total of 92 complete S gene reference sequences from FCoV strains were downloaded from the GenBank database (Supplementary Table 2). In addition, FCoV related coronaviruses like canine coronavirus (CCoV), porcine respiratory coronavirus (PCoV) and transmissible gastroenteritis virus (TGEV) reference S gene sequences were also retrieved (data not shown) to ensure a diverse representation of coronaviruses for comparative analysis. Partial S gene nucleotide analysis showed deletion of some nucleotides in all sequences obtained in the present study. The deletions were observed between positions 22959–22961, 22968–22973, 22983–22991, 23045–23049 and 23055–23057 compared to the reference strain NC_002306.3. Interestingly, these deletions were absent in all reference strains belonging to FCoV type 2 (FIP). Out of 68 FCoV strains detected in this study, 42 formed a separate cluster along with reference strain AY307020. This cluster was further divided into several small sub-clusters (Figure 2) FCoV strains detected from ascitic fluid or effusions were closely related to FCoV strains detected from rectal swabs, intestine, brain and liver tissues.

### Recombination analysis

Using RDP5 analysis of the partial spike gene sequence compared to other members of the genus alpha-coronaviruses-1 showed multiple recombination signals and events. A total of 17 unique recombinant events and 36 recombination signals were observed by using different recombination methods (RDP, GENECOV, MaxChi, Chimaera, Bootscan, SiScan, 3Seq) in RDP5 (Supplementary Figure 2A). These recombination signals might have been caused by an evolutionary process other than recombination. Recombination events shown by three or more recombination methods were used for analysis. The RDP5 analysis revealed that the recombinant isolate of FCoV (PQ561610 FCoV-128) was detected by six detection methods (Supplementary Figure 2B). Based on the multiple sequence alignment of 75 partial S gene sequences, analysis using the RDP5 software showed the beginning breakpoint of the recombination event for PQ561610 FCoV-128 was at position 225 (confidence 99% CI) in this alignment (182 without gaps). The ending breakpoint of the recombination event was found at position 14 in the alignment (5 without gaps). The major parent in this recombination event (PQ561619 FCoV-142) was 81.8% similar to the recombinant (PQ561610 FCoV-128) (Supplementary Figures 3A,B). Similarly, the recombinant PQ561624 FCoV-162 showed 94.5% identity to the major parent with the beginning breakpoint of the recombination event at nucleotide 652 in this alignment (585 without gaps) and ending breakpoint of the recombination event was found at position 87 in alignment (77 without gaps) (Supplementary Figures 2C,D). Interestingly, a FCoV type 1 field isolate from Netherlands (JN183882.1) was found to be the possible major parent for PQ561615 FCoV-133 isolate from Istanbul, Türkiye with the beginning breakpoint of the recombination event at nucleotide 614 in this alignment (551 without gaps) and ending breakpoint of the recombination event was found at position 300 in the alignment (257 without gaps). The major parent in this recombination event (JN183882.1) was 89.4% identical to the recombinant (PQ561615 FCoV-133) These results strongly confirm the presence of recombination events among some of the FCoV strains detected in this study. In contrast, we did not find any recombination event or signals with canine coronavirus (GQ477367.1) or porcine coronavirus (DQ811787.1; DQ811789.2).

## Discussion

FCoV infections have been reported worldwide. The increase in cat population and crowding are important risk factors since FCoV is less frequently seen in housed, stray and feral cats compared to shelter cats (2, 3, 21, 36). The immune status, viral load, undergoing surgery, being subjected to environmental stress like crowding and bad living conditions increase the risk of developing FIP (38). Detection of FCoV antibodies and/or FCoV-RNA in the early stage of infection can be a useful indicator to help minimize the spread of FCoVs in a breeding cattery, multi-cat household and FCoV-free household (16, 17, 33, 55). Therefore, it is important to monitor cats living in multi-cat environments in order to reduce and control FCoV infections.

FCoV prevalence may vary depending on the region/country, inclusion criteria (healthy or ill), selection bias, analytical errors and low specificity of the diagnostic tests. The results of this study indicate that FCoV infections are widespread in cats from Istanbul and this agrees with the results of other studies (27, 30, 31, 56, 57). In the present study, overall seropositivity for FCoV was found to be 90.9%, and was similar over two years (2018 and 2019). However, FCoV seroprevalence was found to be higher in this study compared to a previous study performed in Istanbul (36). This might be associated with an increase in the cat population in recent years and this finding suggests that it will be difficult to find FCoV-seronegative cats in the future. High FCoV seroprevalence (up to 84%) has also been reported in other countries (31, 44, 56, 58). A much lower seroprevalence in chronically ill (19.3%) and healthy cats (10.1%) was reported in Korea (59). The high seroprevalence found in many countries might be attributed to the presence of maternal antibodies but the percentage FCoV seropositivity was also found to be high particularly in this study. Because of the presence of maternal antibodies in younger ages, detection of FCoV RNA by PCR is a better indication of FCoV infection. However, PCR analysis cannot discriminate between FCoV and FIPV at present, although there are some trials aimed at developing specific tests to differentiate them by nucleotide polymorphisms. However, compared to seropositivity, detection of FCoV-RNA in pleural effusion and ascites has a better diagnostic value (42, 60).

In the present study, the overall detection rate of FCoV-RNA in faecal swabs was 37.9% (79/208). Intestinal colonization by FCoV has been investigated by RT-PCR and found to be 37% in Japan (31), 47.5% in Portugal (61), 76.5% in Germany (62) and 84% in Malaysia (30). A high prevalence (84.4%) of FCoV infection in faeces in cat populations in some areas of China has also been reported. They found no significant difference in FCoV-RNA positivity between body cavity effusion from sick cats and faeces from healthy cats (62). In the Fujian Province of China, the overall prevalence of FCoV-RNA positivity in the faeces of cats was 67.9% (63). However, fecal detection is not correlated with ongoing FIP infection (49). Ascitic fluid from 854 cats with suspected FIP was analysed by RT-PCR in Japan and FCoV-RNA was detected in 377 samples (64). In another study performed in Türkiye, 14 (54%) of healthy and sick cats were positive for FCoV-RNA in blood (24).

Results of a retrospective study from 24 American veterinary teaching hospitals showed that FIP prevalence was 0.4% (1,420 cats from 397,182) over a 10-year period (65). The percentage of FCoV-infected cats that subsequently developed FIP was found to be between 8 and 10% in two studies (66, 67). In the present study, the number of FIP cats was most likely about 22 that are PCR positive, and effusions were found as well as histopathological lesions suggestive of FIP. However, limitation of this study is that immunohistochemistry was not performed on these cats died of FIP suspicion.

Several risk factors have been found to be associated with FCoV infection and development of FIP, which include stress, surgery, age, breed, gender, multi-cat environment, different housing conditions and the frequency of litter box disinfection (28–30, 36, 57, 68, 69). While our study identified several significant associations, we caution that some marginal results (e.g., hematological variables) could reflect Type I error. Future replication in larger cohorts with pre-specified hypotheses is warranted.

FCoV infections are usually seen in cats less than 2 years old and especially during the first year of life (1, 28, 31, 36, 49). However, older cats may also be affected (70, 71). In a previous study performed in Istanbul, FCoV serological status was significantly associated with age, FIP serological status and habitat variables. In contrast, age exhibited a bimodal distribution in our study, with cats aged 23–59 weeks showing a lower risk of PCR positivity compared to both younger and older cats. There was no association with age and FCoV-RNA positivity in Malaysian, Australian and Chines studies (28, 30, 63, 69). This could be due to increased cat-to-cat contact in young cats before their immune system reaches maturity, facilitating efficient virus replication and favoring mutation from FECV to FIPV and lack of natural and protective immunity at young ages (49, 63). Young cats may also experience stress due to various factors, including weaning, vaccination, dietary changes, neutering, separation from the queen, and living in a multi-cat environment (36, 55, 72). Stress provokes the release of glucocorticoids, which may cause suppression of cell-mediated immunity and facilitate FCoV replication (49, 72).

No difference in FCoV seroprevalence was found between females and males in previous studies (16, 28, 30, 31, 36, 58, 63, 73). Similar results were found in a previous study in Istanbul (36). In contrast, in Australia and the USA, FCoV prevalence was higher in male cats compare to females (49, 57, 68). Similarly, FIP occurred significantly more often in male cats as reported by others (35, 68, 74). which may be due to regional differences. In Japan, PCR positivity in ascitic fluids was significantly higher in males (51.5%) than in females (35.7%). However, gender was not significantly associated with presence of antibody which indicate the development of FIP in male cats is higher than females (64). In the present study, no significant association was found between gender and FCoV-RNA positivity or seropositivity. At present, there is no biological evidence supporting gender-associated susceptibility and resistance to FCoV. The difference shown in previous studies could be related to males and females having different lifestyles, facilitating FCoV exposure and living in different regions. Alternatively, sex-based differences may be related to androgens, which may negatively affect the immune system, increasing the risk of virus replication (75). However, results of another study indicated that male and female cats had similar risks of developing FIP (47).

Breed was not associated with FCoV seropositivity in either this study or a previous study conducted in Istanbul (36). Multiple international studies have reported higher FCoV seroprevalence in pedigree cats compared to non-pedigree cats (28, 31, 49, 57, 58). In a Japanese study of 17,392 cats, FCoV antibody prevalence was significantly higher in pedigree cats (67%) than in non-pedigrees (31%) (31). In another study, Norwegian forest cats and Birmans had a lower risk of FCoV infection (69). Similarly, FIP disproportionately affects pedigree cats (57, 74, 76). In contrast, no specific breed has been identified as a risk factor for FCoV infection in cats in Germany (12), Fujian Province China (63), and in Türkiye (36). In the present study, 55 of 162 cross breed and 24 of 46 purebred cats were found to be positive for FCoV-RNA and this was statistically significant in cross breed cats. This finding contrasts with the results reported from others since the PCR positivity in ascitic fluids was significantly higher in purebreds (62.2%) than in crossbreds (34.8%) in Japan (64). The reason for this can be because of the higher number of cross breed cats than pure-breed cats analyzed in this study.

The effect of a multi-cat environment and housing density on the prevalence of FCoV has been investigated in many studies. Significantly increased risks have been found for cats living in a multi-cat environment, which could lead to virus mutation and development of FIP. Household cats living alone had the lowest risk of being FCoV seropositive (29, 36, 55, 69). The number of cats in a house hold or cattery was significantly associated with the occurrence of FIP in one study (77) but not in another (14). In another study, the majority of cats (75.7%) analysed shed FCoV at least once (69). Results of a previous study performed in Istanbul indicated that household cats that cohabitated with other cats had a high risk of being FCoV seropositive (36), as has been previously shown (14, 18, 26, 27, 30, 57). In contrast to previous findings, most FCoV-RNA positive cats in our study were household cats. Among these, FCoV-RNA positivity showed significant associations with being home-raised, cohabitation with other cats, and outdoor access. This pattern suggests that environmental factors—particularly outdoor exposure and multi-cat households—may facilitate viral transmission and potentially contribute to FCoV mutation and FIP development. These observations align with established literature documenting higher FCoV prevalence in multi-cat environments (62, 69, 78). A previous Istanbul study reported significantly lower FCoV seroprevalence in stray cats (30%) compared to household cats (57%) (36). While prior research suggests household cats with outdoor access and multi-cat cohabitation are disproportionately susceptible to FCoV infection, our study revealed comparable seroprevalence rates (∼90%) across groups, with no statistically significant differences observed.

FCoV infection typically causes mild to moderate clinical signs, primarily diarrhea and lethargy. In cases progressing to FIP, the disease manifests as either effusive (‘wet’) or non-effusive (‘dry’) forms, with clinical signs reflecting disease severity. The most common FIP symptoms include persistent fever (often fluctuating), anorexia, and profound lethargy. Ocular manifestations of non-effusive FIP may include uveitis and retinitis. Neurological involvement can present with seizures, nystagmus, progressive ataxia, and paresis (2, 3, 21). Our study found significantly higher prevalence rates of fever, lethargy, diarrhea, ascites, and pleural effusion in FCoV-PCR positive cats compared to PCR-negative controls (*p* < 0.05). Notably, pleural effusion showed particularly strong association in seropositive cases. These findings align with previous research that additionally identified weight loss and vomiting as clinical markers significantly associated with FCoV seropositivity (36). Consistent with previous reports (73), weight loss and pleural effusion were identified as predominant clinical signs in FIP cases, often accompanied by ascites, lethargy, and anorexia. Recent findings further characterize the FIP presentation profile, reporting abdominal distension (68%), depression (60%), dehydration (58%), anorexia (53%), and dyspnea (42%) as common manifestations (35).

Although the following hematological and biochemical blood parameters are not specific for FIP, thrombocytopenia, normochromic anemia, lymphopenia particularly in cats with effusions, neutrophilia and microcytosis were frequently reported (3, 36, 70, 79–81). No association was found with anemia and occurrence of effusions in one study (82). However, a difference between FIP and microcytosis has been previously reported (70). A decrease in red blood cell count has been reported but attributed to poor prognosis of FIP (80, 83). In a recent study, lymphopenia developed in 75% of cats with FIP, while 45.5% showed neutrophilia, and 13.6% had monocytosis (35).

Characteristic serum biochemical abnormalities in FIP cases include hyperbilirubinemia, hyperproteinemia, hyperglobulinemia, and hypoalbuminemia (3, 21). Particular diagnostic significance lies in the identification of hyperglobulinaemia accompanied by hypoalbuminaemia or low-to-normal serum albumin (3, 41, 70, 73). In addition, low albumin to globulin (A:G) ratio can have a better diagnostic value than either total serum protein or globulin (3, 36). In the present study, as also reported previously, a decrease in serum A/G ratio was detected in most of the cats suspected of having FIP (3, 42, 73). In a recent study, blood profiles revealed mild anemia, lymphopenia, thrombocytopenia, hypoalbuminemia, hyperglobulinemia, and an albumin to globulin ratio of 0.4 in FIP cases (35).

In the present study, the deletion of specific nucleotides was observed in S gene sequences of FCoV type 1. Spike protein is crucial for the virus’s ability to enter host cells. It plays a significant role in the virus’s infectivity and is a primary target for neutralizing antibodies. The implications of S gene variations in FCoV for virulence, transmission, and strain evolution are complex and can significantly impact disease dynamics. S gene deletions (e.g., in the 3′ end) are associated with the transition from feline enteric coronavirus (FECV, low virulence) to feline infectious peritonitis virus (FIPV, high virulence). Loss of certain regions (e.g., furin cleavage sites) may alter cell tropism, allowing systemic spread and macrophage infection, a hallmark of FIP. In addition, S gene deletions may increase or decrease enteric replication, environmental persistence and cat-to-cat transmission. Furthermore, clustering of S gene variants may reflect immune selection pressure, leading to escape mutants that evade neutralizing antibodies (virus evolution).

This complexity arises from the nature of RNA viruses like FCoV, where genetic variation frequently occurs (5, 10, 73, 84–89). In this study, the nucleotide sequence of the S gene of FCoV showed 81.2 to 99.6% nucleotide identity between them. This identity range suggests a high degree of genetic variation between different FCoV strains. The lower end of this range indicates more divergent strains, while the higher end suggests closely related strains. The finding that all 68 FCoV strains sequenced belonged to FCoV-I indicates that these strains share a common evolutionary lineage, which can provide insights into transmission dynamics, virulence, and potential vaccine development. Similar findings were reported from China by Shi et al. (89).

Several unique recombinant events and recombination signals were observed for some FCoV strains in this study by using seven recombination testing methods. Recombination events are indeed a significant aspect of the evolution of coronaviruses, including those affecting both humans and animals. The spike protein gene, in particular, is a critical region for recombination, as it is responsible for viral entry into host cells and plays a key role in determining the virus’s virulence and host range (13, 87, 90–92). FCoV is known for its ability to undergo recombination, which can significantly impact its assembly, invasion, and pathogenicity. Recombination events between different strains of FCoV can lead to increased genetic diversity. This diversity allows the virus to adapt to changing host environments and immune responses, potentially resulting in more virulent strains. For instance, the transition from the less pathogenic FCoV type I to the more pathogenic type II is believed to involve recombination with a canine coronavirus, which may enhance its ability to infect feline hosts. Recombination plays a crucial role in the pathogenesis of FIP, a severe and often fatal disease caused by a mutant form of FCoV. The recombination events may lead to the generation of a more virulent strain that can evade the host’s immune response, resulting in systemic infection and severe inflammatory responses (2). The ability of FCoV to recombine and create variants that can infect macrophages is critical for the development of FIP (93). The assembly of FCoV involves the formation of viral particles through the interaction of structural proteins. Recombination can affect the genes encoding these proteins, potentially altering the efficiency of viral assembly and the virus’s ability to enter host cells. Recombination can lead to the emergence of viral variants that possess mutations in epitopes recognized by the host immune system. This allows the virus to evade neutralizing antibodies generated from previous infections or vaccinations (2, 94). Such immune escape is particularly relevant in the context of FCoV, where previous exposure to less pathogenic strains does not confer protection against the more virulent forms. The recombination of FCoV can also result in variants that are more adept at transmission between cats. Enhanced transmissibility can lead to outbreaks of FIP in multi-cat environments such as shelters or breeding facilities. The ability to recombine and generate new strains that maintain or enhance transmissibility is a key factor in the epidemiology of FCoV infections. Recombination events may facilitate the adaptation of FCoV to new host species, potentially leading to zoonotic spillover or the emergence of new variants that can infect other animals. This host range expansion can complicate control measures and increase the virus’s impact on feline populations.

All FCoV strains identified in this study phylogenetically clustered with type I variants, with no type II strains detected. This finding aligns with global epidemiological patterns, where type I FCoV demonstrates significantly higher prevalence than type II across most geographic regions (59, 62, 73, 95). Our study revealed substantial genetic diversity among type I FCoV strains circulating in Istanbul’s feline population. This high degree of variability presents significant challenges for infection control and eradication, as divergent viral variants may harbor distinct virulence determinants.

Analyses for virus detection should focus on tissues and effusions presumably containing FIPV-infected macrophages rather than blood since viremia is not always found in FCoV infected cats (81, 96). When FCoV was detected in feces, it was likely to be detected in other organs and tissues, and vice versa (49). Besides, FCoV-RNA can be found in the blood of healthy cats (24). An interesting finding reported by others (49) indicated the high detection rate and viral burdens of FCoV in urine and kidney samples suggesting urine as a convenient and valuable sample for FIP diagnosis. Similarly, a high detection rate of feline morbillivirus in the kidney has been reported (97). However, the association between renal damage and urine positivity needs to be studied in FIP cases.

In this study, qRT-PCR analysis detected FCoV RNA in tissue samples from 33 of 43 postmortem feline cases (76.7%). Among these PCR-positive cats, 22 (66.7%) exhibited gross and/or histopathological lesions consistent with FIP diagnosis. Out of the 22 cats, 12 exhibited the mixed form of the disease (12/22, 54.5%). Although clinical examinations often distinguish between effusive and non-effusive forms of the disease, this finding supports the notion that mixed forms of FIP may be under-reported. This is particularly true when diagnosis is based solely on clinical observations, as postmortem examinations can reveal both granulomatous lesions in organs and effusions in body cavities (55, 93, 98, 99). The number of cats with abdominal effusion was higher than those with thoracic effusion, with the characteristic gross findings predominantly localized in the abdominal cavity, as previously described (1, 100).

The histopathological hallmarks of FIP observed in this study included fibrinous serositis, focal to disseminated granulomas with or without necrosis and, less commonly, perivasculitis. At least two of these findings were present in 22 of the 33 PCR-positive cats. Fibrinous serositis and granulomas were most frequently detected in the liver, followed by the kidneys and the intestines, aligning with previous studies. Perivasculitis was predominantly observed in the liver, followed by lungs, brain and kidneys as described previously (1, 98, 101, 102). Of the 33 cats which were positive for FCoV RNA, 11 did not show macroscopic or microscopic findings specific to FIP. Several factors may explain this discrepancy. One possibility is the high sensitivity of the PCR technique, which may detect FCoV even in the absence of gross and microscopic lesions. Another explanation is that these cats might have been in the early stages of the disease where characteristic lesions had not yet developed. Alternatively, sampling limitations could account for the absence of lesions, as characteristic histopathological findings might be missing in the examined tissues (93, 98, 103). On the other hand, while histopathological findings suggestive of FIP were observed in various organs of 8 cats, PCR testing did not yield positive results. Although histopathological examination can identify lesions indicative of FIP, it is insufficient for a definitive diagnosis. Immunohistochemistry (IHC) improves diagnostic accuracy by detecting FCoV antigen within characteristic lesions, particularly in cases where histopathological findings and PCR results are inconsistent (38, 98, 103). Unfortunately, IHC could not be performed in this study due to project constraints.

In our study, histopathological and PCR findings showed complete diagnostic agreement in 25 cases (22 FIP-positive and 3 FIP-negative). Among the examined tissues (liver, brain, and intestines), the liver demonstrated the highest concordance rate between both diagnostic methods. These results reinforce existing evidence that hepatic tissue provides the most reliable sampling site for FCoV detection when using PCR as a confirmatory diagnostic approach (98). However, recent findings suggest the kidney can also be targeted for sampling to aid diagnosis of FIP (49).

## Conclusion

“Our research provided a comprehensive understanding of FCoV and revealed a higher prevalence of FCoV in cats in Turkey. This study might provide an outline for future research on the development of new vaccines and antiviral therapies. It highlights several critical findings and implications: i: The study identifies a higher prevalence of FCoV in Turkish domestic cats, potentially influenced by factors such as multi-cat environments, population density, or limited preventive measures. This underscores the need for targeted interventions in similar settings; ii: Significant genetic mutations observed could affect viral behavior, such as enhanced transmissibility or pathogenicity, possibly contributing to FIP, a severe outcome of FCoV; iii: Evidence of recombination suggests evolutionary dynamics that may lead to noel variants, complicating disease management. Regular epidemiological surveillance for FCoV infections among domestic and stray cats is needed to gain better insights into viral evolution, risk factors, and transmission dynamics, risk assessments and management strategies.”

## Data Availability

The datasets presented in this study can be found in online repositories. The names of the repository/repositories and accession number(s) can be found in the article/Supplementary material.

## Supplementary material

SUPPLEMENTARY FIGURE 1 Histologic lesions from necropsied cats positive for FCoV RNA by qRT-PCR (Hematoxylin-eosin stain). **(A)** Liver, fibrinous perihepatitis and granulomatous hepatitis. Thick band of fibrin along the serosal surface (star) with few infiltrates of inflammatory cells and granulomas in the parenchyma (arrows). **(B)** Large intestine, fibrinous and granulomatous serositis (star). **(C)** Kidney, granulomatous nephritis. Granulomas with necrosis (stars). Inset: Higher magnification of a granuloma (arrow) composed of macrophages, lymphocytes and plasma cells. **(D)** Brain, perivasculitis, mononuclear perivascular infiltrates (arrows).

SUPPLEMENTARY FIGURE 2 **(A)** Pairwise identity for recombination of PQ561619 FCoV-142. Light grey shading: 99% confidence interval (CI) on breakpoint locations. Dark grey shading: 95% confidence interval (CI) on breakpoint locations. **(B)** The heat map shows the region count matrix of the PQ561619 FCoV-142 along with other members of alpha coronaviruses. **(C)** Pairwise identity for recombination for PQ561624 FCoV-162 Light grey shading: 99% confidence interval (CI) on breakpoint locations. Dark grey shading: 95% confidence interval (CI) on breakpoint locations. Purple shedding: region excluded due to presence of missing data/or recombinationally transferred fragments in PQ561624 FCoV-162 (the recombinant). **(D)** The heat map shows the region count matrix of the PQ561624 FCoV-162 along with other members of alpha coronaviruses.

SUPPLEMENTARY FIGURE 3 The recombination analysis of the partial genome sequences of the FCoV field isolates. **(A)** Summary of the information about recombination events and signals. **(B)** The summary of recombination detection methods, recombinants, major parents and minor parents.

SUPPLEMENTARY TABLE 1 Nucleotide homology analysis between 68 FCoV strains in the present study and 92 references strains. The nucleotide identity ranged from 81.41% to 95.81%. PQ561615 and MW316846 shared the highest nucleotide identity (95.81%) while PQ561614 and DQ848678 shared the lowest nucleotide identity (81.41%) in the nucleotide comparative analysis.

SUPPLEMENTARY TABLE 2 Necropsy and histopathological lesions suggestive of FIP in 43 dead cats. Abbreviations: MB: Mix breed, PB: Pure breed, M: Male, F: Female, U: Unknown, N: Negative, P: Positive, GL: Granulomatous lesions, AE: Abdominal effusion, TE: Thoracic effusion, I: Icterus.
