# Supplementary figures and images for: Phylogenetic, clinical, pathological and epidemiological characterization of feline coronavirus infections in cats, in Istanbul
Source: Front Vet Sci. 2025 Oct 10;12:1645884. doi: 10.3389/fvets.2025.1645884 (PMC12550448; doi:10.3389/fvets.2025.1645884)

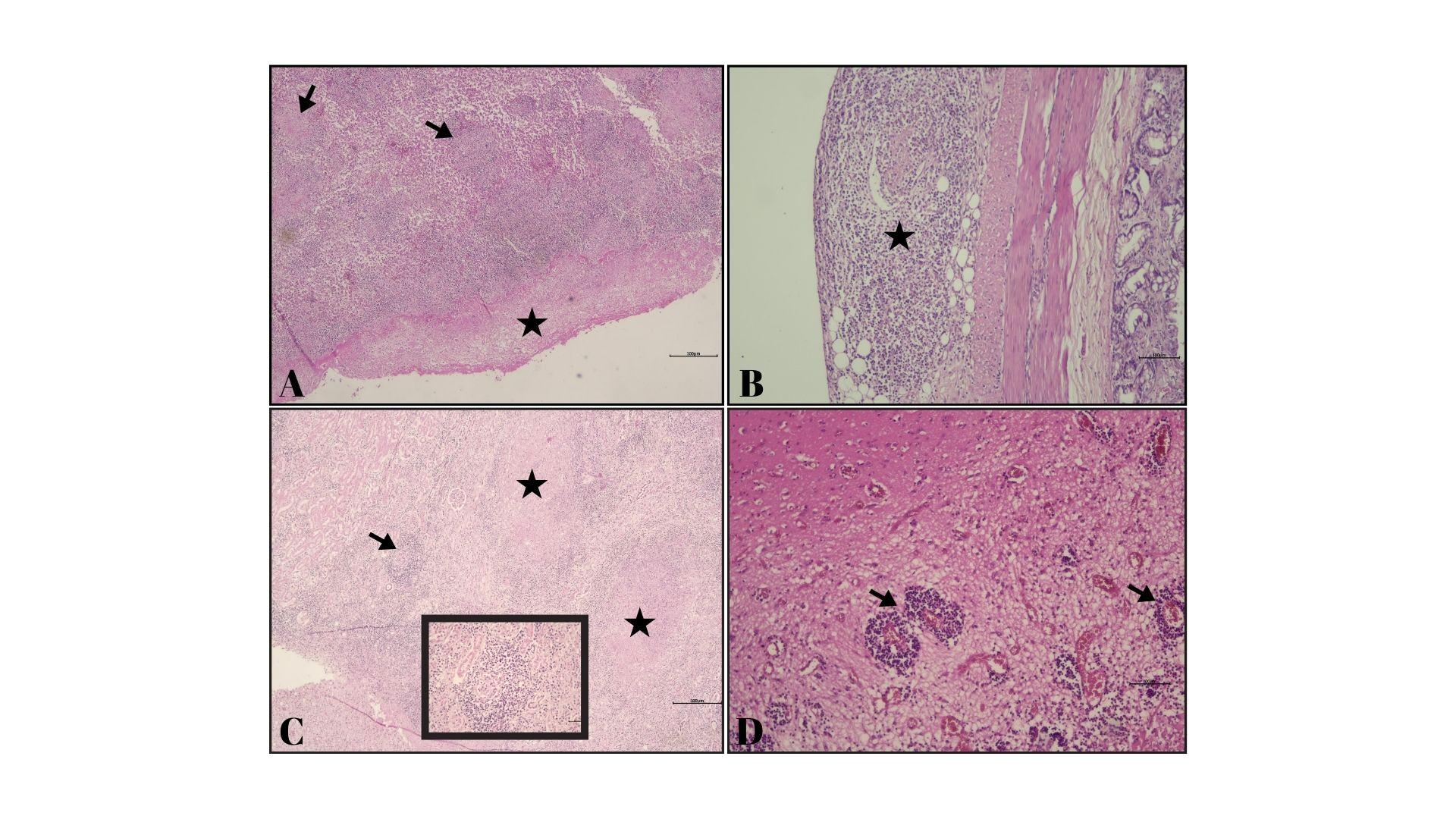

Supplement: SUPPLEMENTARY FIGURE 1 — Histologic lesions from necropsied cats positive for FCoV RNA by qRT-PCR (Hematoxylin-eosin stain). (A) Liver, fibrinous perihepatitis and granulomatous hepatitis. Thick band of fibrin along the serosal surface (star) with few infiltrates of inflammatory cells and granulomas in the parenchyma (arrows). (B) Large intestine, fibrinous and granulomatous serositis (star). (C) Kidney, granulomatous nephritis. Granulomas with necrosis (stars). Inset: Higher magnification of a granuloma (arrow) composed of macrophages, lymphocytes and plasma cells. (D) Brain, perivasculitis, mononuclear perivascular infiltrates (arrows). [file Image_1.PNG]

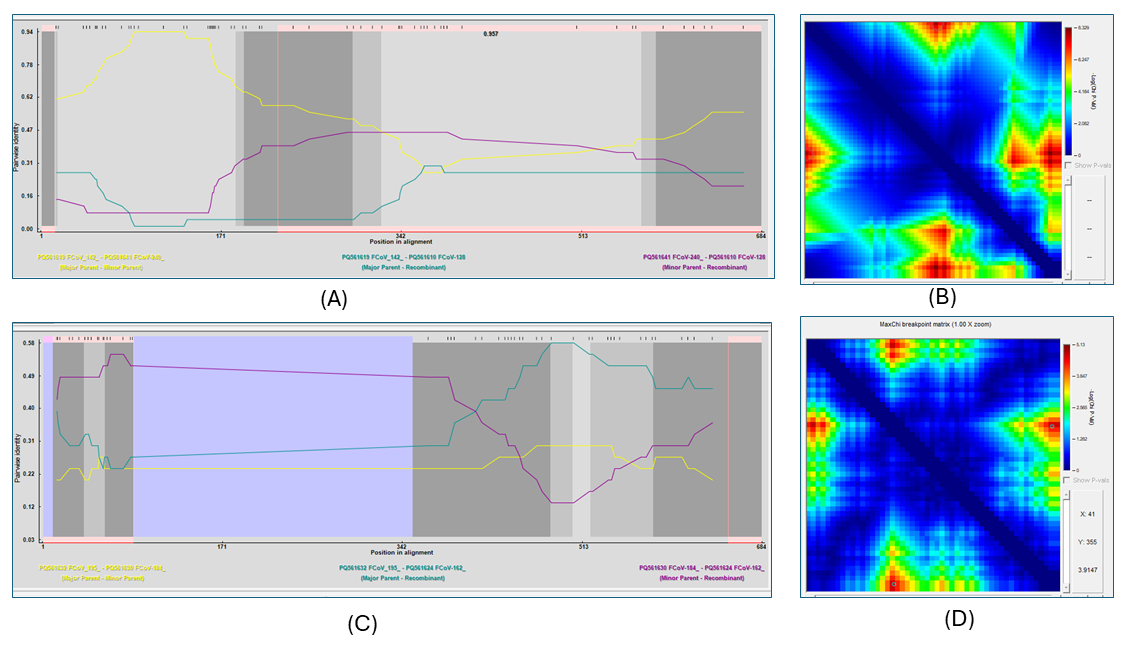

Supplement: SUPPLEMENTARY FIGURE 2 — (A) Pairwise identity for recombination of PQ561619 FCoV-142. Light grey shading: 99% confidence interval (CI) on breakpoint locations. Dark grey shading: 95% confidence interval (CI) on breakpoint locations. (B) The heat map shows the region count matrix of the PQ561619 FCoV-142 along with other members of alpha coronaviruses. (C) Pairwise identity for recombination for PQ561624 FCoV-162 Light grey shading: 99% confidence interval (CI) on breakpoint locations. Dark grey shading: 95% confidence interval (CI) on breakpoint locations. Purple shedding: region excluded due to presence of missing data/or recombinationally transferred fragments in PQ561624 FCoV-162 (the recombinant). (D) The heat map shows the region count matrix of the PQ561624 FCoV-162 along with other members of alpha coronaviruses. [file Image_2.PNG]

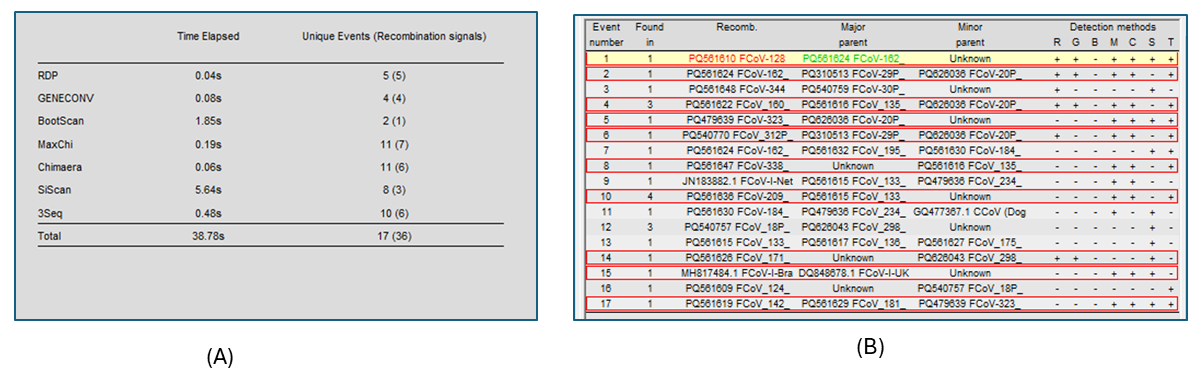

Supplement: SUPPLEMENTARY FIGURE 3 — The recombination analysis of the partial genome sequences of the FCoV field isolates. (A) Summary of the information about recombination events and signals. (B) The summary of recombination detection methods, recombinants, major parents and minor parents. [file Image_3.PNG]
