# Supplementary material for: Phylogenetic, clinical, pathological and epidemiological characterization of feline coronavirus infections in cats, in Istanbul
Source: Front Vet Sci. 2025 Oct 10;12:1645884. doi: 10.3389/fvets.2025.1645884 (PMC12550448; doi:10.3389/fvets.2025.1645884)
Supplement: Supplementary file 2 [file Table_2.DOCX]

| Cat No | Species, Age (months), Gender | Gross findings suggestive of FIP | | Histopathologic findings suggestive of FIP | | | PCR | | |
|---|---|---|---|---|---|---|---|---|---|
| | | | **Histopathologic findings** | Liver | Brain | Intestine | Liver | Brain | Intestine |
| 1 | Mix, 3, M | EMLN | LP hepatitis; catarrhal enteritisD3:D37D3:D42D3:D46 | N | N | N | N | N | P |
| 2 | Mix, 3, M | N | Hepatocellular degeneration; necrotic enteritis; interstitial pneumonia; LA in the spleen; necrotic pancreatitis | N | N | N | N | N | P |
| 3 | Mix, 3, M | N | Interstitial pneumonia | N | N | N | N | P | N |
| 4 | Mix, 5, F | GL | *G hepatitis & vasculitis; LP meningioencephalitis; fibrinous pleuritis* , interstitial pneumonia & *vasculitis; G nephritis & vasculitis* ; LP myocarditis; LP splenitis | P | P | N | P | P | N |
| 5 | Mix, 5, F | EMLN | NSF | N | N | N | P | P | P |
| 6 | Mix, 9, M | N | Necrotic enteritis; interstitial pneumonia & smooth muscle hyperplasia | N | N | N | N | N | P |
| 7 | PB, 7, F | AE, GL | *Fibrinous perihepatitis & vasculitis; cerebral meningitis & vasculitis; necrotic enteritis & G serositis in the intestines* ; interstitial pneumonia; *fibrinous perisplenitis* | P | P | P | P | N | P |
| 8 | Mix,U, M | GL, EMLN | *G hepatitis & vasculitis; cerebral meningitis* ; interstitial pneumonia; *G nephritis & vasculitis; fibrinous perisplenitis* | P | P | N | P | N | N |
| 9 | PB, 3, M | N | Hemorrhage in the liver; catarrhal enteritis; *fibrinous pleuritis,* interstitial pneumonia & *vasculitis, fibrinous pericarditis & LP epicarditis* ; LA in the spleen. | N | N | N | P | N | N |
| 10 | PB, 120, M | AE, GL, I, EMLN | *Fibrinious perihepatitis, G hepatitis, vasculitis; cerebral meningitis;* catarrhal enteritis; *fibrinous pleuritis, G pneumonia & vasculitis;* glomerulonephritis & tubular necrosis, necrotic myocarditis; LP splenitis | P | P | N | P | N | N |
| 11 | PB, 6, F | EMLN | LP hepatitis; *G serositis in the intestines; e* mphysema in the lungs; LA in spleen | N | N | P | P | P | P |
| 12 | PB, 3, M | AE, GL | Hepatocellular degeneraton; *G serositis & necrotic enteritis;* interstitial pneumonia and vasculitis; LA in the spleen | N | N | P | P | N | N |
| 13 | PB, 3, F | N | Necrotic enteritis; interstitial pneumonia; tubular degeneration in kidneys; necrotic myocarditis; LP splenitis | N | N | N | N | N | P |
| 14 | Mix, 8, F | AE, GL, EMLN | *G hepatitis* ; necrotic enteritis; interstitial pneumonia & smooth muscle hyperplasia; LA in the spleen | P | N | N | P | N | N |
| 15 | PB, 3, M | N | Necrotic hepatitis; necrotic enteritis; interstitial pneumonia; LA in the spleen & LP splenitis | N | N | N | P | N | N |
| 16 | Mix, 5, F | AE, TE, GL, EMLN | *Necrotic hepatitis; G serositis in the intestines; fibrinous pleuritis* & interstitial pneumonia; *fibrinous serositis &* interstitial nephritis; LP myocarditis; LA in the spleen | N | N | P | N | N | P |
| 17 | Mix, 36, F | TE, EMLN | Perivasculitis & demyelination in the brain; *fibrinous pleuritis,* smooth muscle hyperplasia & vasculitis in the lungs; tubular degeneration in the kidneys; LP myocaditis; LA in the spleen | N | N | N | N | N | N |
| 18 | Mix, U, F | TE, I, EMLN | Hepatocellular degeneration & *LP perivasculitis in the liver; cerebral meningitis;* necrotic enteritis; *fibrinous pleuritis* & interstitial pneumonia; interstitial nephritis & focal necrosis; epicarditis & myocarditis; LA in the spleen & LP splenit | P | P | N | N | N | N |
| 19 | PB, U, F | AE, TE | Catarrhal enteritis; edema in lungs | N | N | N | N | N | N |
| 20 | Mix, U, F | AE, TE | *Fibrinous perihepatitis & LP perivasculitis in liver; LP perivasculitis &* demyelination in the brain; *fibrinous serositis & G nephritis* | P | P | N | N | N | N |
| 21 | Mix, 72, F | EMLN | Catarrhal enteritis; interstitial pneumonia; glomerulonephritis | N | N | N | N | N | N |
| 22 | PB, 3, M | EMLN | Necrotic hepatitis; *LP perivasculitis in brain* ; necrotic enteritis; interstitial pneumonia; *fibrinous serositis & interstitial nephritis;* LP splenitis | N | N | N | P | N | P |
| 23 | Mix, U, M | AE, GL | *PG hepatitis & vasculitis; cerebral meningitis;* necrotic enteritis; *fibrinous pleuritis;* tubular degeneration in kidneys; LA in spleen | P | P | N | N | P | P |
| 24 | Mix, 3, M | GL | *G hepatitis & vasculitis* ; smooth muscle hyperplasia in the lungs; tubular degeneration in the kidneys; LA in the spleen | P | N | N | N | N | N |
| 25 | U, U, F | N | Catarrhal enteritis | N | N | N | N | N | N |
| 26 | Mix, 12, M | AE, GL | *G hepatitis & vasculitis; cerebral meningitis* & demyelination; catarrhal enteritis; interstitial pneumonia; interstitial nephritis; necrotic myocarditis; LA in the spleen | P | P | N | N | N | N |
| 27 | Mix, 6, M | EMLN | *Fibrinous perihepatitis, G hepatitis & vasculitis* ; edema & emphysema in the lungs; interstitial nephritis; pericarditis & epicarditis; LA in the spleen | P | N | N | N | N | N |
| 28 | Mix, 12, M | AE, EMLN | *Fibrinous perihepatitis, G hepatitis & vasculitis; cerebral meningitis; catarrhal enteritis & fibrinous serositis; fibrinous pleuritis & vasculitis;* interstitial nephritis; *fibrinous pericarditis; fibrinious perisplenitis & LP splenitis* | P | P | P | P | P | P |
| 29 | PB, 30, F | AE, TE, GL, I, EMLN | *Fibrinous perihepatitis, G hepatitis & vasculitis; cerebral meningitis & vasculitis; G serositis in the intestines; fibrinous pleuritis & vasculitis in the lungs; fibrinous serositis & G nephritis; fibrinous pericarditis & myocarditis; fibrinous peri* | P | P | P | P | P | P |
| 30 | Mix, U, F | AE, TE, GL, I, EMLN | *Fibrinous perihepatitis, G hepatitis & vasculitis; cerebral meningitis; G serositis in the intestines; fibrinous pleuritis, PG penumonia & vasculitis;* interstitial nephritis; fibrinous serositis & lymphoid atrophy in the spleen | P | P | P | P | P | P |
| 31 | U, U, F | TE, GL | *G hepatitis & vasculitis; cerebral meningitis & perivasculitis; catarrhal enteritis & fibrinous serositis in the intestines; fibrinous pleuritis, G pneumonia & vasculitis; tubular necrosis & G nephritis; fibrinous pericarditis & myocarditis; fibrin* | P | P | P | P | P | N |
| 32 | Mix, 3, M | AE, TE, GL | *Fibrinous perihepatitis, G hepatitis & vasculitis; fibrinous serositis in the intestines; fibrinous pleuritis, G pneumonia & vasculitis; G myocarditis;* LP splenitis | P | N | P | N | N | N |
| 33 | Mix, 3, F | N, EMLN | Catarrhal enteritis; tubular degeneration in the kidneys; LA in spleen | N | N | N | N | P | P |
| 34 | PB, 3, F | N | Parasitic enteritis | N | N | N | P | P | P |
| 35 | Mix, 3, M | N | Necrotic enteritis; interstitial pneumonia | N | N | N | P | P | P |
| 36 | PB, 3, F | AE, GL | *Fibrinous perihepatitis, G hepatitis & vasculitis;* necrotic enteritis; interstitial pneumonia; *fibrinous serositis & vasculitis in the kidneys* | P | N | N | P | P | N |
| 37 | Mix, 18, M | EMLN | *G hepatitis & vasculitis; cerebral meningitis & perivasculitis* ; necrotic enteritis; interstitial pneumonia; tubular degeneration in the kidneys | P | P | N | P | N | P |
| 38 | PB, 9, M | AE, GL, EMLN | *Fibrinous perihepatitis; LP enteritis & fibrinous serositis* ; hemorrhage in the lungs; glomerulonephritis; lymphoid atrophy in the spleen; LP pancreatitis | P | N | P | P | N | P |
| 39 | PB, 3, M | EMLN | *Fibrinous perihepatitis, G hepatitis & vasculitis; necrotic enteritis & fibrinous serositis* | P | N | P | P | N | P |
| 40 | Mix, 3, F | N | Interstitial pneumonia; tubular degeneration in kidneys; hyaline degeneration in heart; LA in the spleen | N | N | N | N | N | P |
| 41 | PB, 3, F | N | *Hepatocellular degeneration & vasculitis; cerebral meningitis & perivasculitis;* necrotic enteritis; LP splenitis | P | P | N | P | P | P |
| 42 | PB, 3, M | AE | *Fibrinous perihepatitis & vasculitis; cerebral meningitis* ; necrotic enteritis; interstitial pneumonia; interstitial nephritis & acute tubular necrosis; LP epicarditis & myocarditis; necrotic splenitis | P | P | N | P | P | N |
| 43 | Mix, 8, F | GL, EMLN | *Fibrinous perihepatitis, G hepatitis & vasculitis; necrotic meningioencephalitis; G serositis in the intestines; fibrinous pleuritis; G nephritis & vasculitis; fibrinous pericarditis; fibrinious perisplenitis & LP splenitis; fibrinous peripancreatiti* | P | P | P | P | P | P |
